# Associations of discretionary screen time with mortality, cardiovascular disease and cancer are attenuated by strength, fitness and physical activity: findings from the UK Biobank study

**DOI:** 10.1186/s12916-018-1063-1

**Published:** 2018-05-24

**Authors:** Carlos A. Celis-Morales, Donald M. Lyall, Lewis Steell, Stuart R. Gray, Stamatina Iliodromiti, Jana Anderson, Daniel F. Mackay, Paul Welsh, Thomas Yates, Jill P. Pell, Naveed Sattar, Jason M. R. Gill

**Affiliations:** 1 BHF Glasgow Cardiovascular Research Centre, Institute of Cardiovascular and Medical Sciences, College of Medical, Veterinary and Life Sciences, University of Glasgow, Glasgow, G12 8TA UK; 2 Institute of Health and Wellbeing, University of Glasgow, Glasgow, G12 8RZ UK; 3 National Institute for Health Research (NIHR) Leicester-Loughborough Diet, Lifestyle and Physical Activity Biomedical Research Unit (BRU), Leicester Diabetes Centre, Leicester General Hospital, Gwendolen Road, Leicester, LE5 4PW UK; 4 Diabetes Research Centre, University of Leicester, University Road, Leicester, LE1 7RH UK

**Keywords:** Mortality, Cardiovascular, Screen time, Screen-time, Physical activity, Fitness, Strength

## Abstract

**Background:**

Discretionary screen time (time spent viewing a television or computer screen during leisure time) is an important contributor to total sedentary behaviour, which is associated with increased risk of mortality and cardiovascular disease (CVD). The aim of this study was to determine whether the associations of screen time with cardiovascular disease and all-cause mortality were modified by levels of cardiorespiratory fitness, grip strength or physical activity.

**Methods:**

In total, 390,089 participants (54% women) from the UK Biobank were included in this study. All-cause mortality, CVD and cancer incidence and mortality were the main outcomes. Discretionary television (TV) viewing, personal computer (PC) screen time and overall screen time (TV + PC time) were the exposure variables. Grip strength, fitness and physical activity were treated as potential effect modifiers.

**Results:**

Altogether, 7420 participants died, and there were 22,210 CVD events, over a median of 5.0 years follow-up (interquartile range 4.3 to 5.7; after exclusion of the first 2 years from baseline in the landmark analysis). All discretionary screen-time exposures were significantly associated with all health outcomes. The associations of overall discretionary screen time with all-cause mortality and incidence of CVD and cancer were strongest amongst participants in the lowest tertile for grip strength (all-cause mortality hazard ratio per 2-h increase in screen time (1.31 [95% confidence interval: 1.22–1.43], *p* < 0.0001; CVD 1.21 [1.13–1.30], *p* = 0.0001; cancer incidence 1.14 [1.10–1.19], *p* < 0.0001) and weakest amongst those in the highest grip-strength tertile (all-cause mortality 1.04 [0.95–1.14], *p* = 0.198; CVD 1.05 [0.99–1.11], *p* = 0.070; cancer 0.98 [0.93–1.05], *p* = 0.771). Similar trends were found for fitness (lowest fitness tertile: all-cause mortality 1.23 [1.13–1.34], *p* = 0.002 and CVD 1.10 [1.02–1.22], *p* = 0.010; highest fitness tertile: all-cause mortality 1.12 [0.96–1.28], *p* = 0.848 and CVD 1.01 [0.96–1.07], *p* = 0.570). Similar findings were found for physical activity for all-cause mortality and cancer incidence.

**Conclusions:**

The associations between discretionary screen time and adverse health outcomes were strongest in those with low grip strength, fitness and physical activity and markedly attenuated in those with the highest levels of grip strength, fitness and physical activity. Thus, if these associations are causal, the greatest benefits from health promotion interventions to reduce discretionary screen time may be seen in those with low levels of strength, fitness and physical activity.

**Electronic supplementary material:**

The online version of this article (10.1186/s12916-018-1063-1) contains supplementary material, which is available to authorized users.

## Background

Low levels of physical activity [1] and high levels of sedentary behaviour (overall sitting and discretionary television viewing and computer use) [2–4] both have strong associations with a number of adverse health outcomes, including mortality and cardiovascular disease (CVD). There is also strong evidence that low levels of cardiorespiratory fitness [5] and muscular strength [6–9] are associated with similar adverse health outcomes. It has been suggested that the associations of physical activity and sedentary behaviour with health outcomes are largely independent [1, 2, 10]. However, a recent meta-analysis indicated that the adverse effects of sitting time and television (TV) viewing on mortality were not observed in individuals with high levels of physical activity [10]. This observation indicates that the risks associated with sedentary behaviour are not ubiquitous, since individuals with low physical activity experience the greatest adverse effects. Using data from UK Biobank, which includes almost 500,000 participants with data on grip strength and over 60,000 participants with data on cardiorespiratory fitness, we recently reported that the adverse association of low levels of physical activity with mortality and CVD events are substantially stronger in individuals with low levels of grip strength and/or cardiorespiratory fitness. This impies that the benefits of physical activity may be greatest amongst individuals with lower levels of functional capacity, but relatively modest in those with already high levels of functional capacity, who were at low risk irrespective of physical activity levels [9]. This has implications for public health guidance, as it suggests that specifically targeting those with low fitness and strength to increase their physical activity levels may be an effective approach to reduce population risk [9]. We hypothesised that a similar pattern would be evident for discretionary screen-time behaviours such as TV viewing and personal computer (PC) screen time, with the adverse effects of high levels of screen time being greatest in those with low levels of strength, fitness and physical activity [10].

The aim of this study was, therefore, to determine whether the associations of screen time (TV viewing plus PC screen time) with mortality and CVD and cancer incidence was moderated by grip strength, cardiorespiratory fitness and physical activity using data from UK Biobank—a large prospective population-based study. Screen time (i.e. time spent viewing a TV or PC screen during leisure time), which represents a substantial proportion of total sedentary behaviour during leisure time [11, 12], was used as a measure of screen-time-related behaviours.

## Methods

### Study design

Between April 2007 and December 2010, UK Biobank recruited 502,655 participants (5.5% response rate), aged 40–69 years, from the general population [13]. Participants attended one of 22 assessment centres across England, Wales and Scotland [14, 15], where they completed a touch-screen questionnaire, had physical measurements taken and provided biological samples, as described in detail elsewhere [14, 15]. In this prospective population-based study, all-cause mortality, CVD incidence and mortality, and cancer incidence and mortality were the main outcomes. The duration of overall discretionary screen time (TV viewing plus leisure PC screen time), and TV viewing and leisure PC screen time separately were the exposures of interest. Socio-demographic factors (age, sex, ethnicity, Townsend deprivation index, professional qualifications, income and employment), smoking status, body mass index (BMI) categories, physical activity, grip strength, sleep duration and dietary intake were treated as potential confounders, as were systolic blood pressure, medication history for glucose, cholesterol and blood pressure as well as prevalent diabetes and hypertension at baseline. Grip strength, cardiorespiratory fitness and physical activity were treated as potential effect modifiers. To minimise potential reverse causality, all analyses were conducted using a landmark analysis excluding events occurring in the first 2 years of follow-up. Moreover, participants with baseline medical diagnoses of depression, chronic obstructive pulmonary disease (COPD), chronic asthma, chronic liver diseases, alcohol problems, substance abuse, eating disorders, schizophrenia, cognitive impartment, Parkinson’s disease, dementia, chronic pain syndrome, heart diseases or cancer were excluded (*n* = 103,755).

### Procedures

Date of death was obtained from death certificates held by the National Health Service (NHS) Information Centre (England and Wales) and the NHS Central Register Scotland (Scotland). Date and cause of hospital admissions were identified via record linkage to Health Episode Statistics (England and Wales) and to the Scottish Morbidity Records (Scotland). Detailed information regarding the linkage procedure can be found at http://biobank.ctsu.ox.ac.uk/crystal/label.cgi?id=2000. At the time of analysis, mortality data were available up to 31 January 2016. The mortality analysis was, therefore, censored at this date or date of death if this occurred earlier. Hospital admission data were available until 31 March 2015, resulting in disease-specific analyses being censored at this date, or the date of hospital admission or death if these occurred earlier. Follow-up information on cancer was obtained via linkage to three routine administrative databases, death certificates, hospital admissions and cancer registrations, with complete follow-up available until 31 March 2015. CVD was defined as a hospital admission or death with ICD-10 code I05-I89.9. All-cause cancer was defined as an ICD-10 code of C0.0-C9.9, D3.7-9 or D4.0-8.

At baseline assessment, screen time and physical activity were recorded among participants recruited from August 2009 using a touch-screen, self-completed questionnaire. Participants were asked: ‘In a typical day, how many hours do you spend watching TV?’ They were also asked about time spent using a computer: ‘In a typical day, how many hours do you spend using the computer? (Do not include using a computer at work)'. For this study, we derived a discretionary screen-time variable that combined TV viewing and leisure PC screen time in hours per day.

Physical activity was based on the International Physical Activity Questionnaire (IPAQ) short form [16], with participants reporting the frequency and duration of walking and moderate and vigorous activity undertaken in a typical week [16]. Data were analysed in accordance with the IPAQ scoring protocol [17] and total physical activity was computed as the sum of walking and moderate and vigorous activity, measured as metabolic equivalent (MET, hours/week). Participants were excluded from the analyses if they recorded implausible values, that is, if the sum of their total physical activity, sleeping time and total screen time exceeded 24 h (*n* = 705 participants were excluded) [9].

Grip strength was assessed using a Jamar J00105 hydraulic hand dynamometer and the mean of three measurements for each hand were used. Grip strength was measured in kilograms. Fitness test data were introduced into UK Biobank from August 2009, so these data are available only in a subgroup of 74,836 participants. In these individuals, cardiorespiratory fitness was assessed using a sub-maximal 6-min incremental ramp cycle ergometer test with workload calculated according to age, height, weight, resting heart rate and sex, and heart rate monitored via a four-lead electrocardiogram, as previously reported, with the aim of achieving a final work rate of 50% of predicted maximal power [9]. Tests were terminated if heart rate exceeded 75% of the age-predicted maximum. In individuals with systolic blood pressure between 160 and 179 mmHg or diastolic blood pressure between 95 and 109 mmHg or who answered ‘yes’ or ‘unsure’ to the question ‘Has a doctor ever said that you have a heart condition and should only do physical activity recommended by a doctor’, the test protocol was modified to achieve a final work rate of 35% of predicted maximal power (*n* = 8932). Fitness was not measured in individuals who were not able to walk or cycle unaided for 10 min, were pregnant or had high blood pressure (systolic blood pressure ≥180 mmHg or diastolic blood pressure ≥110 mmHg) (*n* = 358) or if the equipment failed (*n* = 643). The work rate at maximal heart rate was estimated by extrapolating the pre-exercise heart rate (i.e. at work rate zero watts) and the heart rate and work rate at the end of the test to the age-predicted maximal heart rate (208 – 0.7 × age) [18] assuming a linear relationship [19]. The linear nature of the work rate vs heart rate relationship means that the estimated maximal work rate for an individual should be independent of the work rate achieved during the exercise test. Maximal oxygen uptake (i.e. at maximal heart rate) was estimated from the regression equation for the relationship between work rate and oxygen uptake (oxygen uptake (in ml.kg^− 1^.min^− 1^) = 7 + (10.8 × work rate (in watts))/body mass (in kilograms)) [20] and then expressed in terms of maximal MET (where 1 MET ≡ 3.5 ml.kg^− 1^.min^− 1^).

Dietary information was collected via a self-reported dietary questionnaire (Oxford WebQ) [21, 22]. Participants were asked how many portions of specified foods they generally ate. Subjective sleep duration was obtained by asking: ‘About how many hours sleep do you get in every 24 hours?’ Based on the answer, we derived a categorical sleep duration variable (short sleeper <7 h.day^− 1^, normal sleeper 7–9 h.day^− 1^ or long sleeper >9 h.day^− 1^). Area-based socioeconomic status was derived from postcode of residence using the Townsend score, which is derived from census data on housing, employment, social class and car availability [23]. Other socio-demographic information such as employment (paid employment, retired, unable to work, unemployed, student and other), professional qualifications (college or university, A or O levels, GCSE, CSEs or equivalent levels) and income (<£18,000, £18,000–29,999, £30,000–51,999, £52,000–100,000 and >£100,000) were self-reported at baseline. Age was calculated from dates of birth and baseline assessment. Ethnicity was self-reported and smoking status was categorised into never, former and current smoking. Medical history (physician diagnosis of long-standing illness, depression, stroke, angina, myocardial infarction, hypertension, cancer and diabetes) and medication history (for diabetes, cholesterol and hypertension) were collected from the self-completed baseline assessment questionnaire. Height, body weight and waist circumference were measured by trained nurses during the baseline assessment. Body composition (percentage body fat) was measured using standardised bio-impedance protocols. BMI was calculated as (weight/height^2^) and the World Health Organization criteria [24] were used to classify BMI into underweight <18.5, normal weight 18.5–24.9, overweight 25.0–29.9 and obese ≥30.0 kg.m^− 2^. Central obesity was defined as waist circumference >88 cm for women and >102 cm for men. Further details of these measurements can be found in the UK Biobank online protocol (http://www.ukbiobank.ac.uk/resources/) and our supplementary material. The numbers of participants with missing data for covariates are described in Additional file 1: Table S1.

### Statistical analyses

The associations between hours of overall discretionary screen time, TV viewing and PC screen time per day and health outcomes were explored using Cox-proportional hazard models with years of follow-up as the time scale. Analyses were performed for the following outcomes: all-cause mortality and CVD and cancer incidence (fatal and non-fatal combined) and mortality. All analyses were performed as a landmark analysis with follow-up commenced 2 years after recruitment and including participants who were event-free at this time. In addition, participants with comorbidities (depression, COPD, chronic asthma, chronic liver diseases, alcohol problems, substance abuse, eating disorders, schizophrenia, cognitive impartment, Parkinson, dementia, chronic pain syndrome, heart diseases and cancer) at baseline were excluded from all analyses (*n* = 103,755).

Firstly, the durations of discretionary screen time, TV viewing and PC screen time in hours per day were treated as continuous variables and hazard ratios (HR) were calculated per 1-h increment. Linearity was explored with fractional polynomial models for each exposure, with no evidence for deviation from linearity. Each exposure was rounded to the nearest hour. Multiplicative interactions between the screen-time exposures and sex were investigated by fitting the relevant parameters into the model. For these analyses, we ran four incremental models that included an increasing number of covariates. Model 0 included age, sex, ethnicity, deprivation index, professional qualifications, income and employment as covariates. Model 1 was adjusted for lifestyle factors including smoking, physical activity, grip strength, categories of sleep duration, dietary intake (alcohol, fruit and vegetables, red meat, processed meat and oily fish intake). Model 2 was adjusted for model 1 plus BMI categories. Model 3 was adjusted for model 2 plus systolic blood pressure, prevalent diabetes, hypertension and medication for diabetes, hypertension, and cholesterol. Finally, model 4 was equivalent to model 3 but participants who reported to be ex-smokers (*n* = 173,104) or current smokers (*n* = 52,990) were excluded from the analysis.

To investigate whether grip strength, cardiorespiratory fitness or physical activity moderated the associations between screen-time exposures and health outcomes, participants were stratified into age- and sex-specific tertiles for grip strength, cardiorespiratory fitness and physical activity (Additional file 1: Tables S2–S4), and all screen-time exposures were classified into the following categories: <2 h, 2–3 h, 4–5 h and >5 h. Significant interactions of physical activity, fitness and strength with screen-time exposures on health outcomes were tested by fitting an interaction term between the exposure of interest and the modifier factors coded as ordinal variables (i.e. TV viewing category × physical activity tertiles). To illustrate the interaction effect, we used ordinal coding with the referent group being the lowest category for the duration of screen time (<2 h.day^− 1^) and the highest tertile for grip strength, fitness or physical activity. These interaction analyses were adjusted for model 3 mentioned above, but physical activity and grip strength were removed as covariates and used as interaction factors.

The proportional hazard assumption was checked by tests based on Schoenfeld residuals. All analyses were performed using statistical software STATA 14 (StataCorp LP).

## Results

Of the 502,655 participants recruited since August 2009, after excluding participants in a landmark analysis with follow-up commencing 2 years after recruitment and participants who self-reported comorbidities at baseline, we included 391,089 participants with available data for discretionary screen time, grip strength and physical activity (Additional file 1: Table S1). Valid cardiorespiratory fitness and screen-time data were available for a subset of 59,068 participants [9]. The median follow-up period was 5.0 years (interquartile range 4.3 to 5.7), commencing 2 years after baseline, for mortality outcomes and 4.2 years for CVD and cancer incidence (interquartile range 3.5 to 4.7). Over the follow-up period, 7420 participants died and there were 22,210 CVD and 23,464 cancer events, of which 2198 and 4606 were fatal, respectively.

The main characteristics of the participants by categories of screen time are summarised in Table 1. In summary, individuals in the highest group for overall screen time (>5 h.day^− 1^) were more likely to be from the most deprived tertile (with lower income, lower professional qualifications and more likely to be retired, unemployed or unable to work because of disability or sickness) compared with the lowest group (<2 h.day^− 1^). Moreover, individuals in this higher screen-time category had a higher prevalence of current smoking, obesity and comorbidities, including diabetes and hypertension as well as higher prevalence of being on medication for hypertension and higher cholesterol, compared with the lowest group (<2 h.day^− 1^). They had a higher BMI, waist circumference and percentage body fat, had a higher intake of processed meat and lower intake of fruit and vegetables, and had lower levels of physical activity, fitness and grip strength in comparison to those in the lowest screen-time group (Table 1). Similar patterns were observed when participants were stratified by TV-viewing categories (Additional file 1: Table S5), but not for PC screen-time categories (Additional file 1: Table S6). Compared to individuals in the lower PC screen-time category (<2 h.day^− 1^), those in the higher category (>5 h.day^− 1^) were more likely to have college or university degrees, be in a higher income group and be currently employed. They had higher fitness and grip strength but lower physical activity levels and lower medication use. No major differences were observed across PC screen categories for dietary intake, adiposity, obesity and comorbidities (Additional file 1: Table S6).

**Table 1:** Cohort characteristics by overall discretionary screen-time categories

	Screen-time categories (h.day^− 1^)			
	<2	2–3	4–5	>5
Socio-demographics				
Total *n*	65,374	204,470	95,877	25,368
Women, *n* (%)	38,977 (59.6)	110,399 (54.0)	51,460 (53.7)	12,519 (49.4)
Age (years), mean (SD)	54.0 (8.0)	55.5 (8.1)	57.8 (7.8)	56.8 (8.2)
Deprivation index quintiles, *n* (%)				
Lower	22,447 (34.3)	74,332 (36.4)	31,308 (32.7)	6643 (26.2)
Middle	21,683 (33.2)	70,242 (34.4)	32,612 (34.0)	7515 (29.6)
Higher	21,244 (32.5)	59,896 (29.3)	31,957 (33.3)	11,210 (44.2)
Professional qualifications, *n* (%)				
College or university degree	33,304 (55.8)	72,395 (40.7)	20,217 (28.2)	5872 (33.2)
A levels/AS levels or equivalent	7943 (13.3)	25,182 (14.2)	9120 (12.7)	2136 (12.1)
O levels/GCSEs or equivalent	10,408 (17.4)	45,118 (25.4)	23,125 (32.3)	5115 (28.9)
CSEs or equivalent	2230 (3.7)	11,517 (6.5)	6666 (9.3)	1641 (9.3)
NVQ or HND or HNC or equivalent	2840 (4.8)	13,182 (7.4)	7388 (10.3)	1881 (10.6)
Other professional qualifications	2984 (5.0)	10,603 (6.0)	5178 (7.2)	1042 (5.9)
Income categories, *n* (%)				
Less than £18,000	7366 (12.8)	28,761 (16.1)	22,871 (28.5)	7808 (36.8)
£18,000 to £29,999	11,344 (19.7)	44,273 (24.8)	23,569 (29.4)	5047 (23.8)
£30,000 to £51,999	15,613 (27.1)	52,221 (29.3)	19,823 (24.7)	4066 (19.2)
£52,000 to £100,000	16,782 (29.2)	42,731 (24.0)	11,718 (14.6)	3277 (15.4)
Greater than £100,000	6432 (11.2)	10,364 (5.8)	2180 (2.7)	1025 (4.8)
Employment status, *n* (%)				
In paid employment or self-employed	48,674 (75.2)	134,628 (66.4)	46,746 (49.2)	11,612 (46.3)
Retired	11,970 (18.5)	56,500 (27.9)	40,443 (42.6)	9862 (39.3)
Looking after home and/or family	2194 (3.4)	5578 (2.8)	2701 (2.8)	681 (2.7)
Unable to work because of sickness or disability	722 (1.1)	2180 (1.1)	2310 (2.4)	1605 (6.4)
Unemployed	662 (1.0)	2435 (1.2)	2117 (2.2)	1089 (4.3)
Doing unpaid or voluntary work	344 (0.5)	907 (0.5)	378 (0.4)	117 (0.5)
Full-time or part-time student	203 (0.3)	522 (0.3)	256 (0.3)	112 (0.5)
Ethnicity, *n* (%)				
White	60,997 (93.3)	193,700 (94.7)	90,686 (94.6)	23,229 (91.6)
South Asian	1806 (2.8)	4095 (2.0)	1580 (1.7)	622 (2.5)
Black	1072 (1.6)	2994 (1.5)	1935 (2.0)	854 (3.4)
Chinese	267 (0.4)	721 (0.4)	312 (0.3)	108 (0.4)
Mixed background / others	1232 (1.9)	2960 (1.5)	1364 (1.4)	555 (2.2)
Smoking status, *n* (%)				
Never	41,168 (63.2)	118,540 (58.2)	50,242 (52.6)	12,551 (49.7)
Previous	18,901 (29.0)	67,082 (32.9)	34,420 (36.0)	8815 (34.9)
Current	5087 (7.8)	18,207 (8.9)	10,870 (11.4)	3889 (15.4)
Obesity-related markers				
BMI, mean (SD)	25.7 (4.2)	27.1 (4.5)	28.2 (4.8)	28.89 (5.4)
BMI categories, *n* (%)				
Underweight (< 18.5)	566 (0.9)	952 (0.5)	280 (0.3)	107 (0.4)
Normal weight (18.5–24.9)	31,288 (48.1)	70,487 (34.6)	24,335 (25.5)	5781 (23.0)
Overweight (25.0 to 29.9)	24,428 (37.5)	88,950 (43.7)	42,855 (44.9)	10,374 (41.3)
Obese (≥30.0)	8788 (13.5)	43,274 (21.3)	27,941 (29.3)	8875 (35.3)
Waist circumference (cm), mean (SD)	85.3 (12.3)	89.2 (12.9)	92.2 (13.3)	94.38 (14.2)
Central Obesity, *n* (%)	13,362 (20.5)	60,575 (29.7)	37,674 (39.4)	11,327 (44.9)
% Body fat, mean (SD)	29.2 (8.3)	30.8 (8.4)	32.7 (8.4)	32.89 (8.9)
Fitness, Physical activity and Sleep, mean (SD)				
Fitness (METs)	9.7 (3.5)	9.2 (3.4)	8.3 (3.3)	8.3 (3.5)
Grip strength (kg)	31.1 (10.7)	31.5 (11.0)	30.4 (11.2)	30.5 (11.3)
Total physical activity (MET.h.week^−1^)	6.6 (8.9)	6.7 (9.2)	6.3 (9.1)	5.0 (7.8)
TV viewing (h.day^−1^)	0.7 (0.4)	2.3 (0.7)	4.1 (0.9)	5.3 (2.4)
PC screen time (h.day^−1^)	0.8 (0.4)	1.0 (0.8)	1.3 (1.4)	3.0 (3.1)
Screen time (h.day^− 1^)	1.6 (0.6)	3.4 (0.9)	5.4 (1.2)	8.2 (2.0)
Sleep duration (h.day^−1^)	7.1 (0.9)	7.1 (1.0)	7.2 (1.1)	7.1 (1.2)
Dietary intake, mean (SD)				
Total energy (kcal.day^−1^)	2124.2 (636.5)	2115.3 (634.9)	2111.3 (660.9)	2126.5 (736.9)
Protein intake (% of TE)	15.3 (3.4)	15.6 (3.6)	15.7 (3.8)	15.6 (4.0)
Carbohydrates intake (% of TE)	47.7 (8.1)	47.1 (8.1)	47.0 (8.2)	46.9 (8.6)
Total Fat intake (% of TE)	31.9 (6.6)	32.0 (6.7)	32.2 (6.8)	32.4 (7.1)
Saturated intake (% of TE)	12.2 (3.3)	12.3 (3.3)	12.4 (3.4)	12.5 (3.5)
Sugar intake (% of TE)	22.8 (6.9)	22.4 (6.9)	22.3 (7.1)	22.2 (7.5)
Alcohol intake (% of TE)	5.1 (6.2)	5.3 (6.4)	5.2 (6.7)	5.0 (7.0)
Red meat intake (portions.week^−1^)	1.8 (1.4)	1.9 (1.4)	2.0 (1.5)	2.1 (1.7)
Processed meat intake (portions.week^−1^)	1.7 (1.1)	1.9 (1.1)	2.0 (1.1)	2.0 (1.1)
Vegetable and Fruit intake (grams.day^−1^)	350.7 (196.9)	331.3 (189.1)	316.7 (192.7)	308.8 (213.5)
Oily fish (portions.week^−1^)	1.1 (1.1)	1.1 (1.0)	1.1 (1.0)	1.0 (1.1)
Health status				
Diabetes history, *n* (%)	1784 (2.7)	7493 (3.7)	5411 (5.7)	2097 (8.3)
High blood pressure history, *n* (%)	11,565 (17.7)	47,541 (23.3)	28,760 (30.1)	8281 (32.8)
Systolic blood pressure (mmHg), mean (SD)	135.7 (19.4)	139.6 (19.5)	142.6 (19.5)	141.6 (19.8)
Diastolic blood pressure (mmHg), mean (SD)	80.6 (10.7)	82.4 (10.6)	83.5 (10.5)	83.6 (10.8)
Medication for cholesterol or blood pressure, *n* (%)				
None of the above	60,310 (92.3)	184,387 (90.2)	82,102 (85.6)	21,719 (85.6)
Cholesterol-lowering medication	2259 (3.5)	9644 (4.7)	7267 (7.6)	2037 (8.0)
Blood pressure medication	2805 (4.3)	10,439 (5.1)	6508 (6.8)	1612 (6.4)

*BMI* body mass index, *MET* metabolic equivalent, *SD* standard deviation, *TE* total energy intake

The characteristics of individuals by tertiles of physical activity, cardiorespiratory fitness and grip strength are presented in Additional file 1: Tables S7–S9. Correlations between TV viewing and PC screen time were low (*r* = − 0.072). Similarly, the correlation of screen-time exposures with grip strength, cardiorespiratory fitness and physical activity were low (ranging from *r* = − 0.199 to 0.115) (Additional file 1: Table S10).

**Fig. 1:**
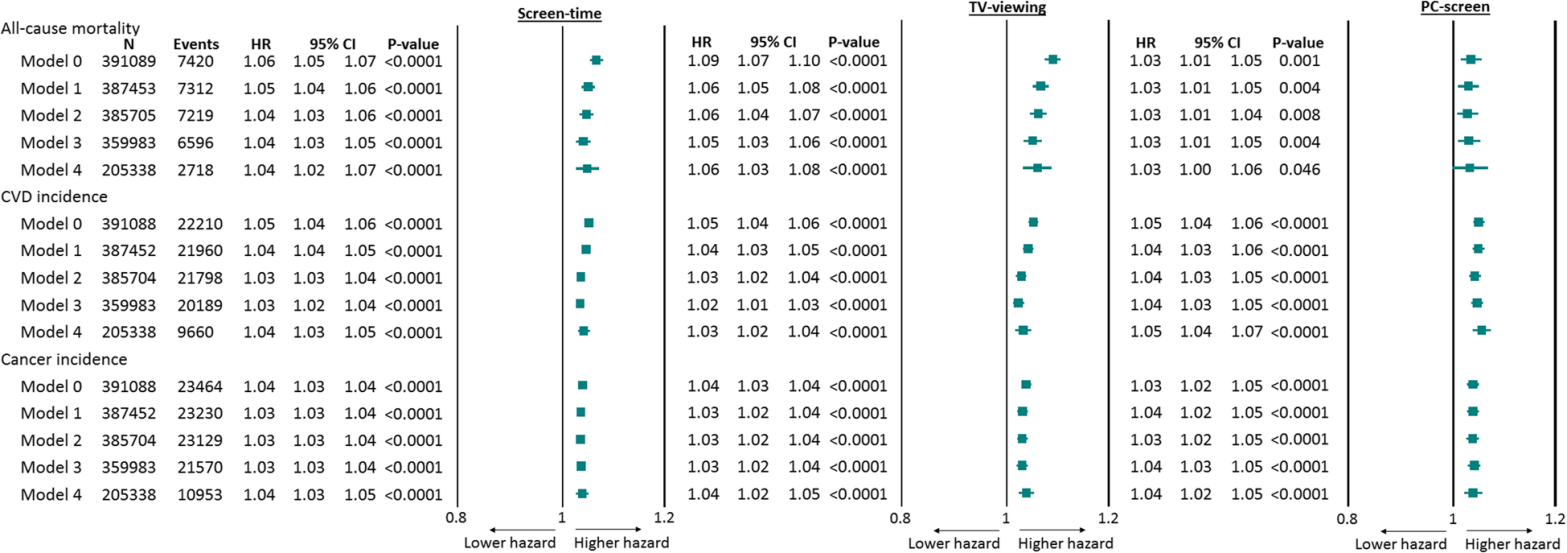
Cox proportional hazard model of the association of 1-h increments in overall discretionary screen time, TV viewing and leisure PC screen time with all-cause mortality and incidence CVD and cancer. Data presented as adjusted hazard ratio (HR) (95% CI) per 1-h increments in discretionary screen time, TV viewing and PC screen time per day. CVD cardiovascular disease, CI confidence interval, HR hazard ratio, PC personal computer, TV television

Overall, there were significant associations of overall discretionary screen time, TV viewing and PC screen time with health outcomes (Fig. 1 and Additional file 1: Figure S1). No significant interactions were found between any of the screen-time exposures and sex for any of the outcomes (data not shown); therefore, analyses were not stratified by sex. Moreover, when BMI categories, diabetes and hypertension prevalence were removed as covariates from the analysis due to their potential mediating role on the outcome, the interactions were not altered (data not shown). The associations of discretionary screen-time exposures and all-cause mortality (HR: 1.06 [95% confidence interval CI: 1.05; 1.07], *p* < 0.0001), incidence of CVD (HR: 1.05 [95% CI: 1.04; 1.06], *p* < 0.0001) and cancer (HR: 1.04 [95% CI: 1.03; 1.04], *p* < 0.0001) were slightly attenuated, but remained associated, after adjustment for the potential confounding effects of socio-demographic characteristics, lifestyle factors (including smoking), physical activity, grip strength and dietary variables and further adjustment for mediators (BMI, diabetes, systolic blood pressure and hypertension prevalence as well as medication for hypertension and cholesterol) (Fig. 1 and Additional file 1: Figure S2). The magnitude of the associations between TV viewing and all-cause mortality (HR: 1.09 [95% CI: 1.07; 1.10], *p* < 0.0001) was slightly higher than those observed for PC screen time (HR: 1.03 [95% CI: 1.01; 1.05], *p* = 0.001); however, the associations between screen-time exposures were similar for CVD and cancer incidence and mortality (Fig. 1 and Additional file 1: Figure S1).

**Fig. 2:**
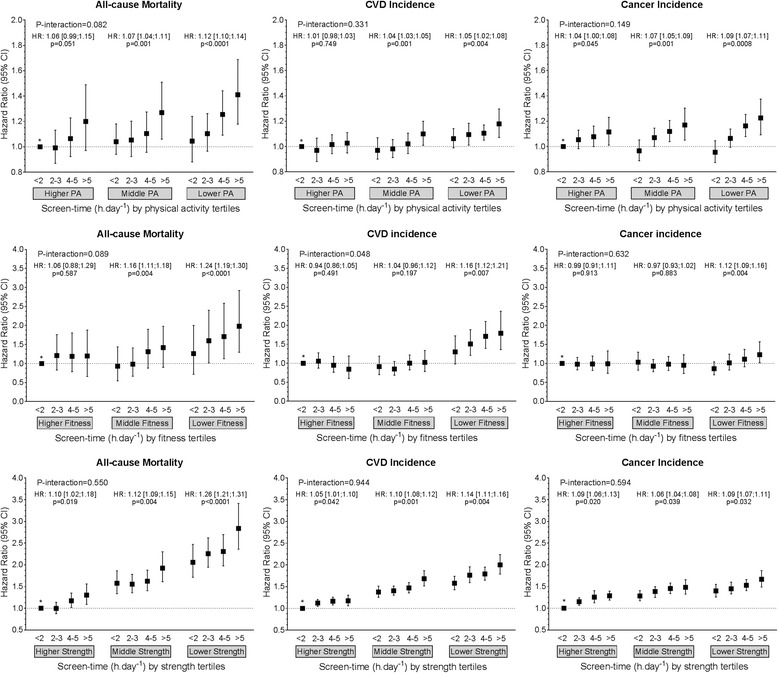
Cox proportional hazard models of the association of overall discretionary screen time with all-cause mortality, and incidence of CVD and cancer by physical activity, fitness and handgrip strength strata. Data are presented as adjusted hazard ratio (HR) (95% CI). Reference category was defined as those participants with < 2 h.day^− 1^ of screen time and who were in the highest tertile for physical activity, fitness or grip strength. Within-tertile HR trends, with *p* values for these trends also shown for each physical activity, fitness and physical activity strata. P-interaction indicates the *p* value for the interaction between screen time and tertile of physical activity, fitness or strength. CVD cardiovascular disease, CI confidence interval, HR hazard ratio, PA physical activity

**Fig. 3:**
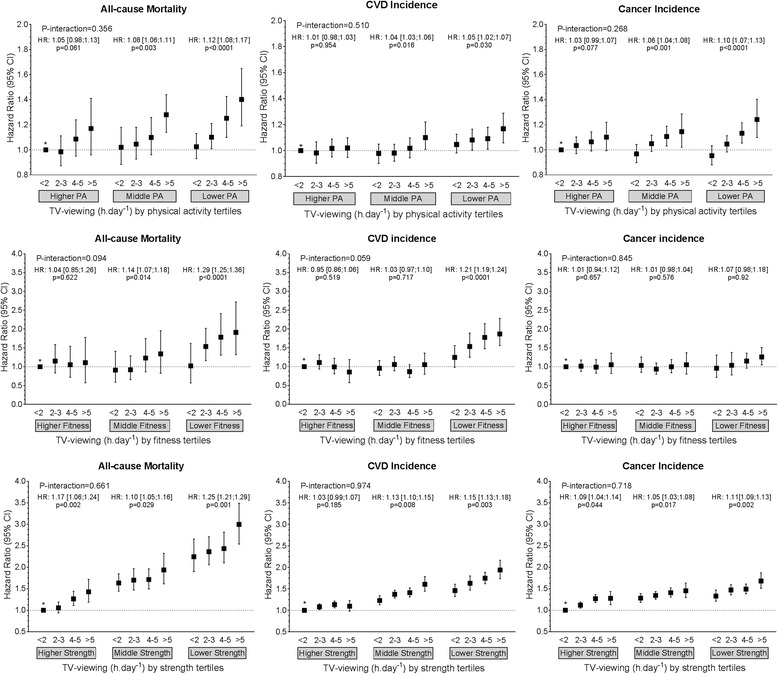
Cox proportional hazard models of the association of TV viewing with all-cause mortality, and incidence of CVD and cancer by physical activity, fitness and handgrip strength strata. Data presented as adjusted hazard ratio (HR) (95%CI). Reference category was defined as those participants with < 2 h.day^− 1^ of TV viewing and who were in the highest tertile for physical activity, fitness or grip strength. Within-tertile HR trends, with *p* values for these trends also shown for each physical activity, fitness and physical activity strata. Analyses were adjusted for age, sex, ethnicity, deprivation index, professional qualifications, income, employment, smoking status, sleep duration categories, dietary intake (alcohol, red meat, processed meat, fruit and vegetable and oily fish intake), systolic blood pressure, prevalent diabetes, hypertension and medication for diabetes, hypertension, and cholesterol. Analyses were all performed as landmark analysis with follow-up commenced 2 years after recruitment and only including participants who were event-free at this time. Participants with comorbidities at baseline were excluded from all-analysis (*n* = 103,755). P-interaction indicates the *p* value for the interaction between T-viewing and tertile of physical activity, fitness or strength

**Fig. 4:**
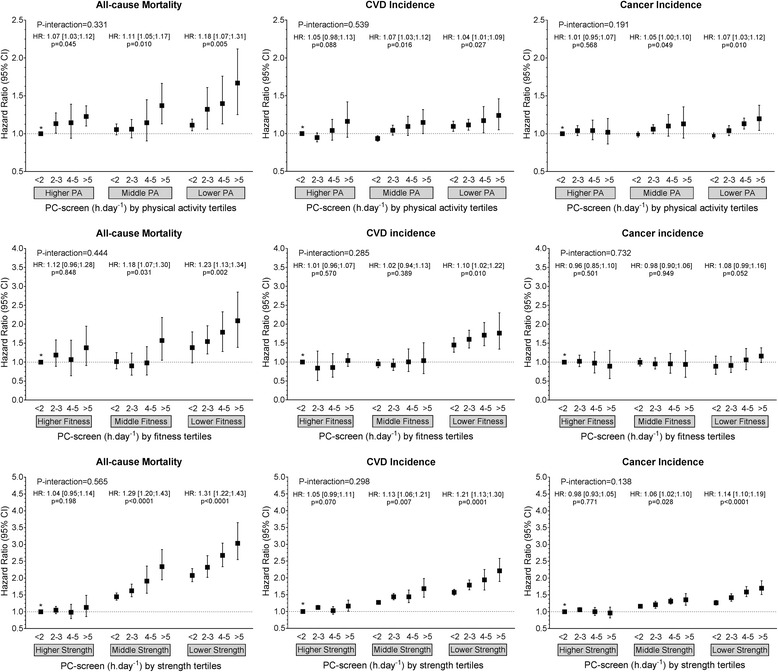
Cox proportional hazard models of the association of leisure PC screen time with all-cause mortality, and incidence of CVD and cancer by physical activity, fitness and handgrip strength strata. Data presented as adjusted hazard ratio (HR) (95%CI). Reference category was defined as those participants with < 2 h.day^− 1^ of PC screen time and who were in the highest tertile for physical activity, fitness or grip strength. Within-tertile HR trends, with *p* values for these trends also shown for each physical activity, fitness and physical activity strata. Analyses were adjusted for age, sex, ethnicity, deprivation index, professional qualifications, income, employment, smoking status, sleep duration categories, dietary intake (alcohol, red meat, processed meat, fruit and vegetable and oily fish intake), systolic blood pressure, prevalent diabetes, hypertension and medication for diabetes, hypertension, and cholesterol. Analyses were all performed as landmark analysis with follow-up commenced 2 years after recruitment and only including participants who were event-free at this time. Participants with comorbidities at baseline were excluded from all-analysis (*n* = 103,755). P-interaction indicates the *p* value for the interaction between PC screen and tertile of physical activity, fitness or strength

No significant interactions were found between any of the screen-time exposures and physical activity, fitness and grip strength for any of the health outcomes, although there was a tendency (*p* < 0.10) for interactions between overall discretionary screen time and fitness for all-cause mortality and CVD incidence and between screen time and physical activity for all-cause mortality (Fig. 2 and Additional file 1: Figures S2–S4 and Tables S11–S13). Nevertheless, when participants were stratified into tertiles for grip strength, physical activity and fitness, the magnitude of HRs for increased risk of adverse health outcomes (all-cause mortality, CVD and cancer incidence) with increasing duration of overall discretionary screen time were numerically highest, and statistically significant, in the subgroup of participants with the lowest levels of grip strength, physical activity or fitness. In contrast, the association of higher screen time with health outcomes in those participants who have higher levels of physical activity, fitness or grip strength was numerically less strong and, with the exception of the association with all-cause mortality in those in the highest tertile of physical activity (HR 1.07 [1.03, 1.13], *p* = 0.045), not statistically significant. For example, considering the association of discretionary screen time with all-cause mortality, the HR per increasing category of screen time was 1.31 (1.22, 1.43) (*p* < 0.0001) for those in the lowest tertile for grip strength, but only 1.04 (0.95, 1.14) (*p* = 0.198) for those in the highest grip strength tertile. When analyses were replicated for TV viewing (Fig. 3 and Additional file 1: Figure S3 and Table S12) and leisure-time PC use (Fig. 4 and Additional file 1: Figure S4 and Table S13), similar trends were observed.

## Discussion

The main novel finding of this study is that the associations between overall discretionary screen time—an index of TV viewing and leisure PC screen time—with all-cause mortality and CVD and cancer incidence and mortality were substantially attenuated by physical activity, cardiorespiratory fitness and grip strength. Our results revealed that, overall, higher levels of screen time were associated with a higher hazard for all-cause mortality and CVD and cancer incidence and mortality, independent of physical activity, grip strength, BMI and other major confounding factors. However, when the cohort was stratified by grip strength, the HRs for mortality, CVD and cancer associated with increasing screen time were almost twice as strong in those with low compared with high levels of grip strength. A similar pattern was observed for physical activity. For cardiorespiratory fitness, the association of increasing screen time with increasing risk for mortality, CVD and cancer was augmented in the least fit population tertile. If these associations are causal, this suggests that the population subgroups with the lowest levels of strength, fitness and physical activity could potentially obtain the greatest benefit from interventions aimed at reducing sedentary behaviours. Conversely, in those with high strength, fitness and physical activity, the adverse effects of prolonged discretionary screen time were attenuated.

Although this study used overall discretionary screen time as one of the exposures of interest, our study also confirms the direction and magnitude of the association of TV viewing and leisure PC screen time with health outcomes [3, 4].

We have recently reported that the associations of physical activity with mortality and CVD events are moderated by both grip strength and cardiorespiratory fitness. The risk associated with low physical activity is substantially greater in those with low strength and fitness, than in those who were strong and fit [9]. Although the present findings indicate that the same is true for overall discretionary screen time and for TV viewing and leisure PC screen time when these were considered separately, the magnitude of the association of TV viewing and PC screen time with health outcomes may not apply to the same extent. Taken together, these findings suggest that individuals with low levels of functional capacity appear to experience the greatest adverse consequences of high levels of screen time and physical inactivity, which, if causal, has implications for public health strategies to reduce mortality, CVD and cancer risk. Current guidelines advocate targeting everyone who has high levels of sedentary behaviour or low physical activity levels with interventions to reduce sitting time and increase physical activity [25]. Our data suggest that targeting such interventions to those with low strength and low fitness could substantially improve our ability to identify individuals who could benefit most from reducing discretionary screen time, which could potentially increase the clinical effectiveness and cost-effectiveness. While fitness testing is relatively difficult to measure in health-care and community settings, grip strength is quick, simple and cheap to measure, and has high reproducibility [26], so could easily be implemented as a screening tool in a variety of settings. The effectiveness of such a targeted approach requires testing in randomised controlled trials.

Conversely, the present data suggest that those with high levels of fitness and grip strength are relatively protected from the adverse association of high levels of screen time with mortality, CVD and cancer risk. High fitness and grip strength are likely to have an innate component since fitness and strength both have relatively high heritability [27, 28]. A number of genes related to fitness and strength have been identified [29], and some individuals with low levels of physical activity are fit and strong [9], but it is also likely that current fitness and strength are influenced by patterns of physical activity earlier in life. There is evidence from animal model studies that artificial selection for high cardiorespiratory fitness leads to a favourable cardio-metabolic risk profile [30] and increased life expectancy [31], suggesting a causal protective effect of high fitness against adverse health outcomes. There are also data from randomised trials of resistance training—which improves strength—that such interventions improve glucose regulation, lipid profile, adiposity and type 2 diabetes risk [32, 33]. Thus, the inverse association between grip strength (which provides a good index of overall muscle limb strength [34]) and mortality/CVD/cancer risk is mechanistically plausible. The present data suggest a high level of functional capacity—as evidenced by high fitness or strength—enables an individual to be able to tolerate high levels of screen time without experiencing the same adverse health consequences as their less fit and strong counterparts. Thus, for individuals who spend a large proportion of their leisure time on screen-related activities (TV viewing and computer use), increasing fitness and strength could conceivably be one way of offsetting this risk. This is a testable hypothesis that warrants testing in a randomised trial.

Our findings also confirm the recent observations in a meta-analysis of a million participants that high levels of physical activity attenuate the adverse effects of prolonged sitting [35]. We extend the findings for a further 502,642 participants to also show that this moderating effect of physical activity on the association between discretionary sedentary behaviour and mortality also applies to CVD and cancer incidence and mortality. Moreover, a recent prospective study reported that 4.3% to 14.9% of premature deaths in the United Kingdom could be avoided through substitution of 30 min.day^− 1^ of total screen time or TV viewing time by discretionary active alternatives, such as DIY and other daily life activities, with the highest potential reduction in mortality to be gained from substituting TV viewing with sport and exercise [4]. This is the largest single study to consider this research question. A key strength of the present investigation is that all participants came from a single well-phenotyped study (UK Biobank), with harmonised data collection and a comprehensive and consistent set of covariates. This eliminates the issue of between-study heterogeneity and enables a more complete adjustment for potential confounding factors than is possible from a meta-analysis.

Higher levels of discretionary screen time, and its subcomponents of TV viewing and leisure PC use, are numerically more strongly associated with adverse health outcomes amongst those with the lowest levels of physical activity, fitness and grip strength. Moreover, there is generally no significant association of increasing screen time with adverse health outcomes amongst the fittest, strongest and most-active tertiles. Despite this, it is important to acknowledge that there were no statistically significant interactions observed when the strengths of these associations were formally statistically compared. This suggests that the power needed to detect such an association is very high and a longer follow-up, to enable more events to accrue, may be needed. On this note, it is important to recognise that the earlier meta-analysis on the association between sitting and mortality according to levels of physical activity—which the present study builds on—did not formally test for interactions and drew its conclusions from numerical differences in the mortality HRs [35].

### Implications of findings

Overall, these data are potentially relevant to guidance and interventions aiming to reduce CVD and mortality risk via behavioural change. Our findings suggest that the deleterious effects of screen time may be greatest amongst those with low levels of strength, fitness or physical activity. Thus, specifically targeting these groups to reduce screen time (and potentially other sedentary behaviours) and/or increase physical activity and functional capacity may be a more effective strategy than the blanket approach of recommending a reduction of sedentary behaviour in all. Notably, it is quick and easy to identify such high-risk groups. The questionnaire used to assess screen time and physical activity in UK Biobank data can be completed relatively quickly (3–5 min). Measurement of grip strength is quick, simple and cheap to administer using a hand grip dynamometer and has high reproducibility [26]. Thus, screening for physical activity level and grip strength could easily be undertaken in routine clinical practice and in community settings to identify individuals for whom reducing sedentary behaviour would be particularly beneficial. Randomised trials to test the effectiveness of such an approach are, therefore, warranted.

### Strengths and limitations

The UK Biobank provided an opportunity to test our research question in a very large, prospective cohort and the main outcome used in this study was collected using a linkage to UK NHS mortality and hospital admission records. Additionally, physical activity, cardiorespiratory fitness and grip strength were assessed using validated methods [9, 17, 22, 36], trained staff and standard operating procedures. In addition, a wide range of potential confounding variables was controlled for in our analyses. These include dietary intake variables, BMI, diabetes and hypertension, which could be on the causal pathway between discretionary screen time and mortality and morbidity outcomes, potentially resulting in over adjustment and, therefore, underestimation of the strength of association for discretionary screen time (and its subcomponents) and the measured outcomes. Fitness was only assessed in a subgroup of the UK Biobank cohort, which limits the power in our analyses stratified by fitness level. In addition, fitness tests were not conducted if it was unsafe to do so and our analyses excluded all participants with comorbidities. It might be expected that such individuals would be more likely to have a low rather than high level of fitness, thus their inclusion could conceivably have obscured or augmented the true association between fitness and the outcome measures due to reverse causality. UK Biobank is relatively representative of the general population with respect to age, sex, ethnicity and deprivation within the age range recruited but is not representative in other regards, such as prevalence of obesity and comorbidities, which may indicate a healthy volunteer selection bias [37]. Whilst this limits the ability to generalise prevalence rates, estimates of the magnitude of associations regarding disease or mortality and disease risk in the current study should nevertheless be generalisable [13, 37, 38]. As is the case for any observational study, causality cannot be confirmed and reverse causality is always a possibility. However, our analyses were conducted by excluding all those with chronic conditions. Although disease and comorbidities existing before the UK Biobank measurement day were self-reported, these self-reported records were based on diseases that have been medically diagnosed. Additionally, we excluded those who died within the first 2 years of follow-up. These approaches help us to minimise the risk of reverse causality influencing our estimates. Another aspect that could confound our findings is the effect of potential mediators, such as BMI, diabetes and hypertension, on the health outcomes. However, we conducted a sensitivity analysis by testing the interaction between health outcomes and screen-time exposures with and without these covariates and the results were not altered. Therefore, we included only a fully adjusted analysis in the study.

Although physical activity was measured by self-report using a validated questionnaire [9, 17, 39], self-reported screen time has not been examined for criterion validity. However, most self-reported instruments have similar validity [40], and the effect estimates reported for screen time in this study were similar to those reported previously in comparable populations using similar adjustment strategies [3, 4, 41]. Misreporting of screen time or physical activity may have attenuated the association between the lifestyle exposures and mortality compared to an objective physical activity measurement [42]. However, this is unlikely to have substantially confounded the differential influence of the exposures on mortality and disease risk across the screen-time or physical activity groups, unless the extent of misreporting of screen time was systematically greater in the groups with the highest levels of physical activity, fitness and grip strength. It is also more feasible to administer a questionnaire rather than an objective measure as a screening tool in routine clinical or community settings [26] to identify individuals for whom increasing physical activity and reducing screen time would be particularly beneficial. The present data suggest that this self-report approach is sufficiently robust to identify differential levels of risk. Although our analyses were adjusted for a number of major confounding factors, we cannot fully discard the potential contribution of unmeasured confounding factors or other proxies of sedentary leisure behaviour in our findings.

## Conclusions

In conclusion, the present data build on existing evidence that a high level of discretionary screen time is a potentially preventable contributor to morbidity and mortality, by demonstrating that this relationship is substantially attenuated by grip strength and cardiorespiratory fitness. We also confirm and extend recent observations that the association between TV viewing and mortality is attenuated by a level of physical activity. This has two potentially important implications for public health. First, interventions to reduce discretionary sedentary behaviours to improve future health outcomes may be more economically and clinically effective if they are targeted at those with low levels of strength, fitness and physical activity. Second, increasing strength and fitness may provide a means of offsetting the potential adverse consequences of high screen time. Both of these warrant testing in future randomised controlled trials.

## Additional file

Additional file 1: **Table S1.** Number of participants with missing data for covariates. **Table S2.** Cut-off points for age- and sex-specific physical activity tertiles. **Table S3.** Cut-off points for age- and sex-specific grip strength tertiles. **Table S4.** Cut-off points for age- and sex-specific fitness tertiles. **Table S5.** Cohort characteristics by categories of TV viewing. **Table S6.** Cohort characteristics by categories of PC screen time. **Table S7.** Cohort characteristics by age- and sex-specific tertiles of total physical activity. **Table S8.** Cohort characteristics by age- and sex-specific tertiles of cardiorespiratory fitness. **Table S9**. Cohort characteristics by age- and sex-specific tertiles of handgrip strength. **Table S10.** Correlation between TV viewing, total physical activity and grip strength. **Figure S1.** Cox proportional hazard model of the association of 1-h increments in screen time, TV viewing and PC screen time with CVD and cancer mortality. **Figure S2.** Cox proportional hazard models of the association of overall discretionary screen time with CVD and cancer mortality by physical activity, fitness and handgrip strength strata. **Figure S3.** Cox proportional hazard models of the association of overall discretionary TV viewing with CVD and cancer mortality by physical activity, fitness and handgrip strength strata. **Figure S4.** Cox proportional hazard models of the association of overall discretionary PC screen time with CVD and cancer mortality by physical activity, fitness and handgrip strength strata. **Table S11.** Cox proportional hazard estimates of the association of overall discretionary screen time with all-cause mortality, CVD and cancer incidence and mortality by physical activity, fitness and handgrip strength strata. **Table S12.** Cox proportional hazard estimates of the association of discretionary TV viewing with all-cause mortality, CVD and cancer incidence and mortality by physical activity, fitness and handgrip strength strata. **Table S13.** Cox proportional hazard estimates of the association of discretionary PC screen time with all-cause mortality, CVD and cancer incidence and mortality by physical activity, fitness and handgrip strength strata. (DOCX 1552 kb)

## Abbreviations

95% CI: 95% confidence interval
BMI: Body mass index
COPD: Chronic obstructive pulmonary disease
CVD: Cardiovascular disease
HR: Hazard ratio
IPAQ: International Physical Activity Questionnaire
MET: Metabolic equivalent
NHS: National Health Service
PC: Personal computer
TV: Television
