# Supplementary material for: Associations of discretionary screen time with mortality, cardiovascular disease and cancer are attenuated by strength, fitness and physical activity: findings from the UK Biobank study
Source: BMC Med. 2018 May 24;16:77. doi: 10.1186/s12916-018-1063-1 (PMC5966877; doi:10.1186/s12916-018-1063-1)
Supplement: Supplementary file 1 — Table S1. Number of participants with missing data for covariates. Table S2. Cut-off points for age- and sex-specific physical activity tertiles. Table S3. Cut-off points for age- and sex-specific grip strength tertiles. Table S4. Cut-off points for age- and sex-specific fitness tertiles. Table S5. Cohort characteristics by categories of TV viewing. Table S6. Cohort characteristics by categories of PC screen time. Table S7. Cohort characteristics by age- and sex-specific tertiles of total physical activity. Table S8. Cohort characteristics by age- and sex-specific tertiles of cardiorespiratory fitness. Table S9. Cohort characteristics by age- and sex-specific tertiles of handgrip strength. Table S10. Correlation between TV viewing, total physical activity and grip strength. Figure S1. Cox proportional hazard model of the association of 1-h increments in screen time, TV viewing and PC screen time with CVD and cancer mortality. Figure S2. Cox proportional hazard models of the association of overall discretionary screen time with CVD and cancer mortality by physical activity, fitness and handgrip strength strata. Figure S3. Cox proportional hazard models of the association of overall discretionary TV viewing with CVD and cancer mortality by physical activity, fitness and handgrip strength strata. Figure S4. Cox proportional hazard models of the association of overall discretionary PC screen time with CVD and cancer mortality by physical activity, fitness and handgrip strength strata. Table S11. Cox proportional hazard estimates of the association of overall discretionary screen time with all-cause mortality, CVD and cancer incidence and mortality by physical activity, fitness and handgrip strength strata. Table S12. Cox proportional hazard estimates of the association of discretionary TV viewing with all-cause mortality, CVD and cancer incidence and mortality by physical activity, fitness and handgrip strength strata. Table S13. Cox proportional hazard estimates of the [file 12916_2018_1063_MOESM1_ESM.docx]

**ADDITIONAL FILES**

**Table S1.** Number of participants with missing data for covariates

| variables | Number of participants with missing data for TV viewing and PC-screen |
| --- | --- |
| BMI | 1808 |
| Waist circumference | 1257 |
| Body fat % | 6417 |
| PC-screen time | 0 |
| TV-viewing | 0 |
| Total physical activity | 0 |
| Fitness | 332021 |
| Handgrip strength | 1510 |
| Macronutrients (totals energy, proteins, carbohydrates, fats and sugar) | 220289 |
| Fruit and vegetable | 0 |
| Red meat | 0 |
| Processed meat | 0 |
| Oily fish | 2152 |
| Alcohol intake frequency | 0 |
| Blood pressure | 24996 |
| Diabetes prevalent | 1079 |
| Hypertension prevalent | 858 |
| Sex | 0 |
| Smoking | 1317 |
| Ethnicity | 0 |
| Deprivation index | 0 |
| Professional qualifications | 64002 |
| Gross income | 53818 |
| Current employment status | 3541 |
| Medication for CVD | 0 |

Data presented as number of participants with missing data.

# **Table S2.** Cut-off point for age and sex –specific physical activity tertile.

| Sex | Age group | Lower | Middle | Higher |
| --- | --- | --- | --- | --- |
| Women | <56 years | <12.5 | 12.5 – 36.5 | >36.5 |
|  | 56 to 65 years | <12.3 | 12.3 – 37.8 | >37.8 |
|  | >65 years | <12.5 | 12.5-40.1 | >40.1 |
| Men | <56 years | <14.4 | 14.4-41.8 | >41.8 |
|  | 56 to 65 years | <14 | 14-42.0 | >42.0 |
|  | >65 years | <15.6 | 15.6-46.2 | >46.2 |

Data presented as METs.hr.week^-1^.

# **Table S3.** Cut-off point for age and sex –specific grip strength tertile.

| Sex | Age group | Lower | Middle | Higher |
| --- | --- | --- | --- | --- |
| Women | <56 years | <23 | 23 – 28 | >28 |
|  | 56 to 65 years | <20 | 20 – 25 | >25 |
|  | >65 years | <18 | 18 – 23 | >23 |
| Men | <56 years | <38 | 38 – 46 | >46 |
|  | 56 to 65 years | <35 | 35 – 42 | >42 |
|  | >65 years | <33 | 33 – 39 | >39 |

Data presented as kg.

# **Table S4.** Cut-off point for age and sex –specific fitness tertile.

| Sex | Age group | Lower | Middle | Higher |
| --- | --- | --- | --- | --- |
| Women | <56 years | <8.0 | 8.0 – 10.1 | >10.1 |
|  | 56 to 65 years | <6.8 | 6.8 – 8.7 | >8.7 |
|  | >65 years | <5.9 | 5.9 – 7.7 | >7.7 |
| Men | <56 years | <10.1 | 10.1 – 12.4 | >12.4 |
|  | 56 to 65 years | <9.2 | 9.2 – 11.4 | >11.4 |
|  | >65 years | <8.1 | 8.1 – 10.5 | >10.5 |

Data presented as METs.

# **Table S5.** Cohort characteristics by categories of TV-viewing

|  | **Screen-time categories (h.day^-1^)** | | | |
| --- | --- | --- | --- | --- |
|  | **<2** | **2-3** | **4-5** | **>5** |
| **Socio-demographics** |  |  |  |  |
| Total n | 83,697 | 199,941 | 87,712 | 17,396 |
| Women, n (%) | 46,251 (55.3) | 108,098 (54.1) | 48,239 (55.0) | 9,199 (52.88) |
| Age (years), mean (SD) | 53.9 (8.0) | 55.5 (8.1) | 58.1 (7.7) | 58.9 (7.7) |
| Deprivation index quintiles, n (%)  Lower  Middle  Higher | 28,478 (34.0)  27,537 (32.9)  27,682 (33.1) | 72,913 (36.5)  68,885 (34.5)  58,143 (29.1) | 28,801 (32.8)  30,020 (34.2)  28,891 (32.9) | 4,043 (23.2)  5,002 (28.8)  8,35 (48.0) |
| Professional qualifications, n (%) |  |  |  |  |
| College or University degree | 45,868 (58.6) | 68,111 (39.3) | 15,673 (24.5) | 1,834 (18.1) |
| A levels/AS levels or equivalent | 10,099 (12.9) | 24,787 (14.3) | 8,158 (12.8) | 1,201 (11.9) |
| O levels/GCSEs or equivalent | 12,407 (15.9) | 45,287 (26.1) | 22,019 (34.4) | 3,675 (36.3) |
| CSEs or equivalent | 2,649 (3.4) | 11,641 (6.7) | 6,385 (10.0) | 1,237 (12.2) |
| NVQ or HND or HNC or equivalent | 3,575 (4.6) | 13,240 (7.6) | 6,933 (10.8) | 1,389 (13.7) |
| Other professional qualifications | 3,632 (4.6) | 10,403 (6.0) | 4,838 (7.6) | 801 (7.9) |
| Income categories, n (%) |  |  |  |  |
| Less than £18,000 | 9,012 (12.0) | 28,311 (16.3) | 21,868 (30.0) | 7,024 (50.4) |
| £18,000 to £29,999 | 14,536 (19.4) | 43,368 (24.9) | 22,133 (30.4) | 3,848 (27.6) |
| £30,000 to £51,999 | 20,426 (27.2) | 51,070 (29.3) | 17,867 (24.5) | 2,147 (15.4) |
| £52,000 to £100,000 | 22,310 (29.7) | 41,737 (24.0) | 9,587 (13.2) | 794 (5.7) |
| Greater than £100,000 | 8,730 (11.6) | 9,766 (5.6) | 1,382 (1.9) | 112 (0.8) |
| Employment status, n (%) |  |  |  |  |
| In paid employment or self-employed | 62,238 (75.0) | 132,327 (66.7) | 41,329 (47.6) | 4,873 (28.4) |
| Retired | 15,344 (18.5) | 54,794 (27.6) | 38,515 (44.3) | 9,181 (53.5) |
| Looking after home and/or family | 2,628 (3.2) | 5,297 (2.7) | 2,507 (2.9) | 622 (3.6) |
| Unable to work because of sickness or disability | 768 (0.9) | 2,178 (1.1) | 2,176 (2.5) | 1,517 (8.8) |
| Unemployed | 999 (1.2) | 2,421 (1.2) | 1,900 (2.2) | 870 (5.1) |
| Doing unpaid or voluntary work | 553 (0.7) | 802 (0.4) | 310 (0.4) | 72 (0.4) |
| Full or part-time student | 423 (0.5) | 469 (0.2) | 160 (0.2) | 33 (0.2) |
| Ethnicity, n (%)  White  South Asian  Black  Chinese  Mixed background / others | 77,905 (93.1)  2,263 (2.7)  1,403 (1.7)  434 (0.5)  1,692 (2.0) | 189,306 (94.7)  4,057 (2.0)  3,055 (1.5)  671 (0.3)  2,852 (1.4) | 83,326 (95.0)  1,353 (1.5)  1,651 (1.9)  242 (0.3)  1,140 (1.3) | 16,123 (92.7)  323 (1.9)  585 (3.4)  45 (0.3)  320 (1.8) |
| Smoking status, n (%)  Never  Previous  Current | 51,833 (62.1)  25,064 (30.0)  6,590 (7.9) | 115,535 (58.0)  65,630 (32.9)  18,150 (9.1) | 45,759 (52.4)  31,600 (36.2)  10,026 (11.5) | 8,079 (46.7)  6,278 (36.3)  2,945 (17.0) |
| **Obesity-related markers** |  |  |  |  |
| BMI, mean (SD) | 25.8 (4.2) | 27.1 (4.5) | 28.3 (4.8) | 29.4 (5.5) |
| BMI Categories, n (%)  Underweight (<18.5)  Normal weight (18.5-24.9)  Overweight (25.0 to 29.9)  Obese (≥30.0) | 683 (0.8)  38,721 (46.5)  32,175 (38.6)  11,730 (14.1) | 885 (0.4)  67,398 (33.8)  87,314 (43.8)  43,560 (21.9) | 252 (0.3)  21,736 (24.9)  39,222 (44.9)  26,079 (29.9) | 73 (0.4)  3,418 (19.9)  6,969 (40.5)  6,747 (39.2) |
| Waist Circumference (cm), mean (SD) | 86.1 (12.5) | 89.4 (12.9) | 92.2 (13.3) | 95.57 (14.3) |
| Central Obesity, n (%) | 17,546 (21.0) | 60,520 (30.4) | 35,156 (40.2) | 8,700 (50.4) |
| % Body fat, mean (SD) | 28.79 (8.3) | 31.0 (8.3) | 33.0 (8.4) | 34.28 (8.8) |
| **Fitness, Physical activity and Sleep**, mean (SD) |  |  |  |  |
| Fitness (METs) | 9.9 (3.5) | 9.1 (3.3) | 8.1 (3.3) | 7.5 (3.3) |
| Grip strength (kg) | 31.9 (10.7) | 31.5 (11.1) | 30.0 (11.1) | 28.8 (11.0) |
| Total physical activity (MET.h.week^-1^) | 6.5 (8.6) | 6.7 (9.2) | 6.4 (9.3) | 5.3 (8.5) |
| TV-viewing (h.day^-1^) | 0.7 (0.4) | 2.5 (0.5) | 4.3 (0.5) | 6.7 (1.3) |
| PC-screen time (h.day^-1^) | 1.4 (1.4) | 1.2 (1.3) | 1.1 (1.3) | 1.0 (1.4) |
| Screen-time (h.day^-1^) | 2.2 (1.5) | 3.6 (1.4) | 5.4 (1.4) | 7.7 (1.9) |
| Sleep duration (h.day^-1^) | 7.1 (0.9) | 7.1 (0.9) | 7.1 (1.1) | 7.2 (1.3) |
| **Dietary intakes**, mean (SD) |  |  |  |  |
| Total energy (Kcal.day^-1^) | 2140.2 (641.4) | 2108.4 (636.5) | 2105.3 (657.5) | 2145.3 (754.8) |
| Protein intake (% of TE) | 15.3 (3.4) | 15.6 (3.6) | 15.7 (3.8) | 15.6 (4.1) |
| Carbohydrates intake (% of TE) | 47.5 (8.2) | 47.1 (8.1) | 47.0 (8.2) | 47.1 (8.3) |
| Total Fat intake (% of TE) | 32.0 (6.7) | 32.0 (6.7) | 32.2 (6.8) | 32.8 (7.1) |
| Saturated intake (% of TE) | 12.2 (3.3) | 12.3 (3.3) | 12.4 (3.3) | 12.8 (3.5) |
| Sugar intake (% of TE) | 22.7 (6.9) | 22.5 (6.9) | 22.2 (7.1) | 22.2 (7.5) |
| Alcohol intake (% of TE) | 5.2 (6.3) | 5.3 (6.5) | 5.1 (6.7) | 4.5 (6.9) |
| Red meat intake (portion.week^-1^) | 1.8 (1.4) | 1.9 (1.4) | 2.1 (1.5) | 2.2 (1.7) |
| Processed meat intake (portion.week^-1^) | 1.7 (1.1) | 1.9 (1.1) | 2.0 (1.1) | 2.1 (1.1) |
| Vegetable and Fruit intake (grams.day^-1^) | 351.3 (199.3) | 330.2 (188.7) | 314.3 (190.7) | 297.2 (211.0) |
| Oily fish (portion.week^-1^) | 1.2 (1.0) | 1.1 (1.0) | 1.1 (1.0) | 1.0 (1.1) |
| **Health status** |  |  |  |  |
| Diabetes history, n (%) | 2,313 (2.8) | 7,478 (3.8) | 5,055 (5.8) | 1,718 (9.9) |
| High blood pressure history, n (%) | 14,751 (17.7) | 47,313 (23.7) | 26,820 (30.7) | 6,502 (37.5) |
| Systolic blood pressure (mmHg), mean (SD) | 135.6 (19.3) | 139.7 (19.5) | 143.0 (19.5) | 144.0 (19.9) |
| Diastolic blood pressure (mmHg), mean (SD) | 80.7 (10.7) | 82.6 (10.6) | 83.6 (10.5) | 84.0 (10.8) |
| Medication for cholesterol or blood pressure, n (%)  None of the above  Cholesterol lowering medication  Blood pressure medication | 78,009 (93.2)  2,521 (3.0)  3,167 (3.8) | 180,024 (90.0)  9,551 (4.8)  10,366 (5.2) | 74,458 (84.9)  7,030 (8.0)  6,224 (7.1) | 14,203 (81.7)  1,826 (10.5)  1,367 (7.9) |

# BMI body mass index; PA physical activity; MET basal metabolic-equivalent; SD standard deviation; n numbers.

**Table S6**. Cohort characteristics by categories of PC-screen

|  | **PC Screen-time categories (h.day^-1^)** | | | |
| --- | --- | --- | --- | --- |
|  | **<2** | **2-3** | **4-5** | **>5** |
| **Socio-demographics** |  |  |  |  |
| Total n | 302,445 | 64,178 | 13,477 | 8,372 |
| Women, n (%) | 175,778 (58.1) | 27,232 (42.4) | 5,343 (39.7) | 3,475 (41.5) |
| Age (years), mean (SD) | 56.0 (8.1) | 56.1 (8.2) | 54.9 (8.2) | 52.3 (7.6) |
| Deprivation index quintiles, n (%)  Lower  Middle  Higher | 106,263 (35.1)  103,432 (34.2)  92,750 (30.7) | 21,320 (33.2)  21,034 (32.8)  21,824 (34.0) | 3,970 (29.5)  4,255 (31.6)  5,252 (39.0) | 2,682 (32.0)  2,615 (31.2)  3,075 (36.7) |
| Professional qualifications, n (%) |  |  |  |  |
| College or University degree | 94,101 (37.9) | 27,085 (47.3) | 6,159 (50.6) | 4,131 (52.4) |
| A levels/AS levels or equivalent | 34,404 (13.9) | 7,370 (12.9) | 1,506 (12.4) | 981 (12.4) |
| O levels/GCSEs or equivalent | 67,463 (27.2) | 12,114 (21.1) | 2,300 (18.9) | 1,540 (19.5) |
| CSEs or equivalent | 17,711 (7.1) | 3,066 (5.4) | 690 (5.7) | 435 (5.5) |
| NVQ or HND or HNC or equivalent | 19,181 (7.7) | 4,463 (7.8) | 927 (7.6) | 532 (6.8) |
| Other professional qualifications | 15,626 (6.3) | 3,203 (5.6) | 588 (4.8) | 264 (3.4) |
| Income categories, n (%) |  |  |  |  |
| Less than £18,000 | 52,500 (20.2) | 10,443 (18.4) | 2,196 (18.3) | 909 (11.9) |
| £18,000 to £29,999 | 65,282 (25.2) | 14,611 (25.7) | 2,635 (22.0) | 1,294 (16.9) |
| £30,000 to £51,999 | 70,475 (27.2) | 15,875 (28.0) | 3,163 (26.4) | 1,991 (26.0) |
| £52,000 to £100,000 | 56,467 (21.8) | 12,371 (22.0) | 3,054 (25.4) | 2,533 (33.1) |
| Greater than £100,000 | 14,623 (5.6) | 3,478 (6.1) | 956 (8.0) | 921 (12.0) |
| Employment status, n (%) |  |  |  |  |
| In paid employment or self-employed | 189,012 (63.0) | 36,170 (57.0) | 8,416 (63.3) | 6,940 (83.6) |
| Retired | 91,887 (30.6) | 21,769 (34.3) | 3,400 (25.6) | 782 (9.4) |
| Looking after home and/or family | 8,787 (2.9) | 1,850 (2.9) | 335 (2.5) | 73 (0.9) |
| Unable to work because of sickness or disability | 4,972 (1.7) | 1,202 (1.9) | 368 (2.8) | 117 (1.4) |
| Unemployed | 3,727 (1.2) | 1,671 (2.6) | 529 (4.0) | 254 (3.1) |
| Doing unpaid or voluntary work | 1,103 (0.4) | 483 (0.8) | 100 (0.8) | 51 (0.6) |
| Full or part-time student | 500 (0.2) | 343 (0.5) | 145 (1.1) | 81 (1.0) |
| Ethnicity, n (%)  White  South Asian  Black  Chinese  Mixed background / others | 287,898 (95.2)  5,603 (1.9)  4,180 (1.4)  821 (0.3)  3,943 (1.3) | 59,371 (92.5)  1,371 (2.1)  1,622 (2.5)  389 (0.6)  1,425 (2.2) | 12,064 (89.5)  392 (2.9)  553 (4.1)  102 (0.8)  366 (2.7) | 7,442 (88.9)  315 (3.8)  299 (3.6)  68 (0.8)  248 (3.0) |
| Smoking status, n (%)  Never  Previous  Current | 174,794 (58.0)  97,824 (32.5)  28,813 (9.6) | 34,387 (53.7)  23,369 (36.5)  6,244 (9.8) | 7,061 (52.6)  4,741 (35.3)  1,634 (12.2) | 4,653 (55.7)  2,670 (32.0)  1,026 (12.3) |
| **Obesity-related markers** |  |  |  |  |
| BMI, mean (SD) | 27.0 (4.6) | 27.9 (4.8) | 28.2 (5.0) | 27.87 (4.9) |
| BMI Categories, n (%)  Underweight (<18.5)  Normal weight (18.5-24.9)  Overweight (25.0 to 29.9)  Obese (≥30.0) | 1,571 (0.5)  107,159 (35.6)  127,869 (42.5)  64,592 (21.5) | 237 (0.4)  18,050 (28.3)  28,270 (44.3)  17,267 (27.1) | 44 (0.3)  3,572 (26.7)  5,844 (43.6)  3,930 (29.4) | 36 (0.4)  2,430 (29.2)  3,546 (42.6)  2,310 (27.8) |
| Waist Circumference (cm), mean (SD) | 88.7 (13.0) | 92.6 (13.3) | 93.6 (13.8) | 92.1 (13.7) |
| Central Obesity, n (%) | 91,254 (30.3) | 22,722 (35.5) | 5,023 (37.4) | 2,840 (34.1) |
| % Body fat, mean (SD) | 31.3 (8.5) | 30.4 (8.6) | 30.3 (8.5) | 30.0 (8.4) |
| **Fitness, Physical activity and Sleep**, mean (SD) |  |  |  |  |
| Fitness (METs) | 8.9 (3.4) | 9.2 (3.4) | 9.1 (3.5) | 9.5 (3.4) |
| Grip strength (kg) | 30.5 (10.9) | 33.0 (11.1) | 33.5 (11.2) | 34.1 (11.2) |
| Total physical activity (MET.h.week^-1^) | 6.7 (9.3) | 6.0 (8.4) | 5.2 (7.3) | 4.4 (5.8) |
| TV-viewing (h.day^-1^) | 2.7 (1.5) | 2.7 (1.6) | 2.7 (1.7) | 2.5 (1.6) |
| PC-screen time (h.day^-1^) | 0.7 (0.5) | 2.2 (0.4) | 4.4 (0.5) | 7.0 (1.5) |
| Screen-time (h.day^-1^) | 3.4 (1.5) | 4.9 (1.6) | 7.1 (1.7) | 9.5 (2.0) |
| Sleep duration (h.day-1) | 7.2 (1.0) | 7.1 (1.0) | 7.0 (1.1) | 6.9 (1.0) |
| **Dietary intakes**, mean (SD) |  |  |  |  |
| Total energy (Kcal.day^-1^) | 2101.0 (633.8) | 2165.8 (661.6) | 2153.8 (714.0) | 2110.9 (727.7) |
| Protein intake (% of TE) | 15.6 (3.6) | 15.4 (3.6) | 15.4 (3.6) | 15.6 (3.9) |
| Carbohydrates intake (% of TE) | 47.3 (8.1) | 46.9 (8.2) | 47.0 (8.5) | 46.7 (9.0) |
| Total Fat intake (% of TE) | 32.0 (6.7) | 32.2 (6.7) | 32.0 (6.9) | 32.0 (7.1) |
| Saturated intake (% of TE) | 12.3 (3.3) | 12.4 (3.3) | 12.4 (3.5) | 12.3 (3.5) |
| Sugar intake (% of TE) | 22.5 (6.9) | 22.3 (7.0) | 22.3 (7.2) | 22.2 (7.5) |
| Alcohol intake (% of TE) | 5.1 (6.4) | 5.5 (6.6) | 5.6 (6.9) | 5.6 (7.1) |
| Red meat intake (portion.week^-1^) | 1.9 (1.4) | 2.0 (1.5) | 2.1 (1.6) | 2.0 (1.6) |
| Processed meat intake (portion.week^-1^) | 1.9 (1.1) | 1.9 (1.1) | 1.9 (1.1) | 1.8 (1.1) |
| Vegetable and Fruit intake (grams.day^-1^) | 329.5 (188.9) | 328.6 (201.8) | 328.9 (211.5) | 331.4 (217.2) |
| Oily fish (portion.week^-1^) | 1.1 (1.0) | 1.1 (1.0) | 1.1 (1.1) | 1.1 (1.1) |
| **Health status** |  |  |  |  |
| Diabetes history, n (%) | 11,820 (3.9) | 3,456 (5.4) | 842 (6.3) | 420 (5.0) |
| High blood pressure history, n (%) | 72,976 (24.2) | 16,809 (26.2) | 3,640 (27.1) | 1,912 (22.9) |
| Systolic blood pressure (mmHg), mean (SD) | 139.9 (19.7) | 139.9 (19.4) | 138.8 (19.0) | 136.5 (18.5) |
| Diastolic blood pressure (mmHg), mean (SD) | 82.3 (10.6) | 82.9 (10.7) | 83.0 (10.8) | 82.7 (10.9) |
| Medication for cholesterol or blood pressure, n (%)  None of the above  Cholesterol lowering medication  Blood pressure medication | 267,578 (88.5)  17,278 (5.7)  17,589 (5.8) | 58,526 (91.2)  2,905 (4.5)  2,747 (4.3) | 12,397 (92.0)  528 (3.9)  552 (4.1) | 7,872 (94.0)  228 (2.7)  272 (3.3) |

BMI body mass index; PA physical activity; MET basal metabolic-equivalent; SD standard deviation; n numbers

# **Table S7.** Cohort characteristics by age and sex-specific tertile of total physical activity

|  | **Physical activity tertiles** | | |
| --- | --- | --- | --- |
|  | **Lower** | **Middle** | **Higher** |
| **Socio-demographics** |  |  |  |
| Total n | 121,796 | 132,919 | 136,374 |
| Women, n (%) | 70,759 (58.1) | 74,869 (56.3) | 67,727 (49.7) |
| Age (years), mean (SD) | 56.2 (7.9) | 56.1 (8.1) | 55.5 (8.3) |
| Deprivation index quintiles, n (%) |  |  |  |
| Lower | 40,640 (33.4) | 40,376 (35.2) | 40,780 (34.7) |
| Middle | 46,753 (33.2) | 45,169 (34.0) | 40,997 (34.1) |
| Higher | 47,337 (33.5) | 46,507 (30.8) | 42,530 (31.2) |
| Professional qualifications, n (%) |  |  |  |
| College or University degree | 37,708 (38.5) | 49,628 (43.4) | 44,452 (38.7) |
| A levels/AS levels or equivalent | 13,582 (13.9) | 15,788 (13.8) | 15,011 (13.1) |
| O levels/GCSEs or equivalent | 26,387 (26.9) | 27,865 (24.4) | 29,514 (25.7) |
| CSEs or equivalent | 6,717 (6.9) | 6,461 (5.7) | 8,876 (7.7) |
| NVQ or HND or HNC or equivalent | 7,404 (7.6) | 7,858 (6.9) | 10,029 (8.7) |
| Other professional qualifications | 6,202 (6.3) | 6,703 (5.9) | 6,902 (6.0) |
| Income categories, n (%) |  |  |  |
| Less than £18,000 | 22,021 (21.8) | 22,041 (18.9) | 22,744 (19.0) |
| £18,000 to £29,999 | 24,808 (24.6) | 28,542 (24.4) | 30,883 (25.8) |
| £30,000 to £51,999 | 27,093 (26.8) | 31,885 (27.3) | 32,745 (27.4) |
| £52,000 to £100,000 | 21,878 (21.7) | 26,810 (23.0) | 25,820 (21.6) |
| Greater than £100,000 | 5,164 (5.1) | 7,535 (6.5) | 7,302 (6.1) |
| Employment status, n (%) |  |  |  |
| In paid employment or self-employed | 75,010 (62.3) | 80,630 (61.2) | 86,020 (63.6) |
| Retired | 35,183 (29.2) | 42,558 (32.3) | 41,034 (30.3) |
| Looking after home and/or family | 3,230 (2.7) | 3,926 (3.0) | 3,998 (3.0) |
| Unable to work because of sickness or disability | 3,967 (3.3) | 1,688 (1.3) | 1,162 (0.9) |
| Unemployed | 2,203 (1.8) | 2,016 (1.5) | 2,084 (1.5) |
| Doing unpaid or voluntary work | 501 (0.4) | 632 (0.5) | 613 (0.5) |
| Full or part-time student | 335 (0.3) | 389 (0.3) | 369 (0.3) |
| Ethnicity, n (%) |  |  |  |
| White | 113,289 (93.0) | 125,890 (94.7) | 129,433 (94.9) |
| South Asian | 3,387 (2.8) | 2,490 (1.9) | 2,226 (1.6) |
| Black | 2,451 (2.0) | 2,140 (1.6) | 2,264 (1.7) |
| Chinese | 526 (0.4) | 456 (0.3) | 426 (0.3) |
| Mixed background / others | 2,143 (1.8) | 1,943 (1.5) | 2,025 (1.5) |
| Smoking status, n (%) |  |  |  |
| Never | 69,069 (57.0) | 76,603 (57.8) | 76,829 (56.5) |
| Previous | 38,600 (31.8) | 44,094 (33.3) | 46,524 (34.2) |
| Current | 13,559 (11.2) | 11,836 (8.9) | 12,658 (9.3) |
| **Obesity-related markers** |  |  |  |
| BMI, mean (SD) | 28.1 (5.2) | 27.0 (4.5) | 26.6 (4.2) |
| BMI Categories, n (%) |  |  |  |
| Underweight (<18.5) | 566 (0.5) | 689 (0.5) | 650 (0.5) |
| Normal weight (18.5-24.9) | 34,476 (28.5) | 46,196 (34.9) | 51,219 (37.7) |
| Overweight (25.0 to 29.9) | 50,297 (41.6) | 57,009 (43.1) | 59,301 (43.6) |
| Obese (≥30.0) | 35,622 (29.5) | 28,516 (21.5) | 24,740 (18.2) |
| Waist Circumference (cm), mean (SD) | 91.8 (13.9) | 89.2 (13.0) | 88.1 (12.5) |
| Central Obesity, n (%) | 48,902 (40.3) | 40,677 (30.7) | 33,359 (24.5) |
| % Body fat, mean (SD) | 33.0 (8.6) | 31.3 (8.3) | 29.3 (8.3) |
| **Fitness, Physical activity and Sleep**, mean (SD) |  |  |  |
| Fitness (METs) | 8.1 (3.3) | 8.9 (3.3) | 9.8 (3.5) |
| Grip strength (kg) | 29.6 (11.0) | 30.8 (10.8) | 32.7 (11.1) |
| Total physical activity (MET.h.week^-1^) | 0.8 (0.7) | 3.9 (1.7) | 14.1 (11.8) |
| TV-viewing (h.day^-1^) | 2.9 (1.69) | 2.6 (1.5) | 2.6 (1.5) |
| PC-screen time (h.day^-1^) | 1.2 (1.4) | 1.2 (1.3) | 1.1 (1.2) |
| Screen-time (h.day^-1^) | 4.1 (2.1) | 3.8 (1.9) | 3.7 (1.8) |
| Sleep duration (h.day-1) | 7.1 (1.1) | 7.1 (1.0) | 7.2 (0.9) |
| **Dietary intakes**, mean (SD) |  |  |  |
| Total energy (Kcal.day^-1^) | 2070.8 (624.9) | 2110.8 (618.4) | 2161.0 (688.6) |
| Protein intake (% of TE) | 15.6 (3.7) | 15.5 (3.5) | 15.5 (3.6) |
| Carbohydrates intake (% of TE) | 46.8 (8.2) | 47.1 (8.0) | 47.5 (8.2) |
| Total Fat intake (% of TE) | 32.5 (6.8) | 32.1 (6.6) | 31.7 (6.7) |
| Saturated intake (% of TE) | 12.6 (3.4) | 12.3 (3.3) | 12.1 (3.3) |
| Sugar intake (% of TE) | 21.9 (7.0) | 22.4 (6.8) | 23 (7.1) |
| Alcohol intake (% of TE) | 5.0 (6.6) | 5.2 (6.4) | 5.3 (6.5) |
| Red meat intake (portion.week^-1^) | 2.0 (1.5) | 1.9 (1.4) | 1.9 (1.4) |
| Processed meat intake (portion.week^-1^) | 1.9 (1.1) | 1.9 (1.1) | 1.8 (1.1) |
| Vegetable and Fruit intake (grams.day^-1^) | 294.9 (179.4) | 328.4 (183.6) | 361.4 (208.4) |
| Oily fish (portion.week^-1^) | 1.0 (1.0) | 1.1 (1.0) | 1.2 (1.1) |
| **Health status** |  |  |  |
| Diabetes history, n (%) | 6,900 (5.7) | 5,370 (4.1) | 4,515 (3.3) |
| High blood pressure history, n (%) | 33,807 (27.9) | 32,520 (24.5) | 29,820 (21.9) |
| Systolic blood pressure (mmHg), mean (SD) | 140.0 (19.7) | 139.6 (19.7) | 139.8 (19.5) |
| Diastolic blood pressure (mmHg), mean (SD) | 83.0 (10.7) | 82.4 (10.7) | 82.1 (10.6) |
| Medication for cholesterol, blood pressure, n (%) |  |  |  |
| None of the above | 105112 (86.3) | 118530 (89.2) | 124876 (91.6) |
| Cholesterol lowering medication | 8548 (7.0) | 7036 (5.3) | 5623 (4.1) |
| Blood pressure medication | 8136 (6.7) | 7353 (5.5) | 5875 (4.3) |

BMI body mass index; PA physical activity; MET basal metabolic-equivalent; SD standard deviation; n numbers

# **Table S8.** Cohort characteristics by age and sex-specific tertile of cardiorespiratory fitness

|  | **Fitness tertiles** | | |
| --- | --- | --- | --- |
|  | **Lower** | **Middle** | **Higher** |
| **Socio-demographics** |  |  |  |
| Total n | 10,543 | 24,056 | 24,469 |
| Women, n (%) | 5,701 (54.1) | 12,966 (53.9) | 13,114 (53.6) |
| Age (years), mean (SD) | 56.4 (8.1) | 56.4 (8.1) | 55.8 (8.2) |
| Deprivation index quintiles, n (%) |  |  |  |
| Lower | 2,646 (25.1) | 3,345 (29.9) | 4,552 (31.9) |
| Middle | 7,181 (31.7) | 8,318 (34.6) | 8,557 (34.4) |
| Higher | 7,816 (43.2) | 8,410 (35.6) | 8,243 (33.7) |
| Professional qualifications, n (%) |  |  |  |
| College or University degree | 2,947 (34.7) | 7,954 (38.8) | 10,598 (48.0) |
| A levels/AS levels or equivalent | 1,185 (13.9) | 2,822 (13.8) | 3,047 (13.8) |
| O levels/GCSEs or equivalent | 2,378 (28.0) | 5,319 (26.0) | 4,790 (21.7) |
| CSEs or equivalent | 732 (8.6) | 1,527 (7.5) | 1,116 (5.1) |
| NVQ or HND or HNC or equivalent | 733 (8.6) | 1,648 (8.0) | 1,346 (6.1) |
| Other professional qualifications | 527 (6.2) | 1,221 (6.0) | 1,188 (5.4) |
| Income categories, n (%) |  |  |  |
| Less than £18,000 | 2,422 (27.4) | 3,919 (18.8) | 2,970 (13.7) |
| £18,000 to £29,999 | 2,427 (27.4) | 5,333 (25.6) | 4,825 (22.2) |
| £30,000 to £51,999 | 2,232 (25.2) | 5,673 (27.2) | 5,876 (27.1) |
| £52,000 to £100,000 | 1,440 (16.3) | 4,625 (22.2) | 5,771 (26.6) |
| Greater than £100,000 | 327 (3.7) | 1,291 (6.2) | 2,281 (10.5) |
| Employment status, n (%) |  |  |  |
| In paid employment or self-employed | 5,790 (55.7) | 14,556 (61.1) | 15,551 (64.1) |
| Retired | 3,412 (32.8) | 7,701 (32.3) | 7,260 (29.9) |
| Looking after home and/or family | 317 (3.1) | 669 (2.8) | 752 (3.1) |
| Unable to work because of sickness or disability | 407 (3.9) | 187 (0.8) | 110 (0.5) |
| Unemployed | 369 (3.6) | 536 (2.3) | 388 (1.6) |
| Doing unpaid or voluntary work | 66 (0.6) | 111 (0.5) | 135 (0.6) |
| Full or part-time student | 44 (0.4) | 66 (0.3) | 55 (0.2) |
| Ethnicity, n (%) |  |  |  |
| White | 8,825 (83.7) | 21,551 (89.6) | 23,220 (94.9) |
| South Asian | 543 (5.2) | 948 (3.9) | 382 (1.6) |
| Black | 789 (7.5) | 840 (3.5) | 263 (1.1) |
| Chinese | 46 (0.4) | 103 (0.4) | 142 (0.6) |
| Mixed background / others | 340 (3.2) | 614 (2.6) | 462 (1.9) |
| Smoking status, n (%) |  |  |  |
| Never | 6,243 (59.5) | 13,981 (58.3) | 13,680 (56.1) |
| Previous | 3,266 (31.1) | 8,048 (33.6) | 8,458 (34.7) |
| Current | 987 (9.4) | 1,956 (8.2) | 2,266 (9.3) |
| **Obesity-related markers** |  |  |  |
| BMI, mean (SD) | 29.8 (5.8) | 27.8 (4.3) | 25.5 (3.6) |
| BMI Categories, n (%) |  |  |  |
| Underweight (<18.5) | 35 (0.3) | 55 (0.2) | 176 (0.7) |
| Normal weight (18.5-24.9) | 2,100 (20.0) | 6,315 (26.3) | 11,693 (47.8) |
| Overweight (25.0 to 29.9) | 4,019 (38.2) | 11,259 (46.8) | 10,035 (41.0) |
| Obese (≥30.0) | 4,366 (41.5) | 6,419 (26.7) | 2,563 (10.5) |
| Waist Circumference (cm), mean (SD) | 95.6 (14.9) | 91.2 (12.5) | 85.6 (11.8) |
| Central Obesity, n (%) | 5,336 (50.6) | 8,859 (36.8) | 4,508 (18.4) |
| % Body fat, mean (SD) | 34.5 (8.8) | 32.4 (8.2) | 28.5 (7.8) |
| **Fitness, Physical activity and Sleep**, mean (SD) |  |  |  |
| Fitness (METs) | 4.2 (2.4) | 8.4 (1.6) | 11.6 (2.5) |
| Grip strength (kg) | 29.0 (11.0) | 30.1 (10.7) | 30.9 (10.6) |
| Total physical activity (MET.h.week^-1^) | 5.4 (8.4) | 6.1 (8.4) | 7.3 (8.6) |
| TV-viewing (h.day^-1^) | 3.1 (1.7) | 2.8 (1.5) | 2.4 (1.4) |
| PC-screen time (h.day^-1^) | 1.3 (1.5) | 1.4 (1.4) | 1.4 (1.3) |
| Screen-time (h.day^-1^) | 4.4 (2.3) | 4.1 (2.0) | 3.7 (1.9) |
| Sleep duration (h.day-1) | 7.1 (1.2) | 7.1 (1.1) | 7.1 (0.9) |
| **Dietary intakes**, mean (SD) |  |  |  |
| Total energy (Kcal.day^-1^) | 2106.6 (741.9) | 2072.4 (639.6) | 2142.9 (642.3) |
| Protein intake (% of TE) | 15.7 (4.1) | 15.7 (3.8) | 15.4 (3.5) |
| Carbohydrates intake (% of TE) | 47.5 (8.6) | 47.3 (8.4) | 47.3 (8.1) |
| Total Fat intake (% of TE) | 32.1 (7.1) | 32.0 (6.9) | 31.7 (6.7) |
| Saturated intake (% of TE) | 12.3 (3.5) | 12.2 (3.4) | 12.0 (3.3) |
| Sugar intake (% of TE) | 22.4 (7.5) | 22.5 (7.2) | 22.7 (6.9) |
| Alcohol intake (% of TE) | 4.7 (6.8) | 5.0 (6.6) | 5.6 (6.5) |
| Red meat intake (portion.week^-1^) | 2.0 (1.6) | 1.9 (1.4) | 1.8 (1.3) |
| Processed meat intake (portion.week^-1^) | 2.0 (1.1) | 1.9 (1.1) | 1.8 (1.1) |
| Vegetable and Fruit intake (grams.day^-1^) | 317.2 (206.7) | 324.0 (200.8) | 344.2 (194.1) |
| Oily fish (portion.week^-1^) | 1.1 (1.1) | 1.1 (1.0) | 1.2 (1.0) |
| **Health status** |  |  |  |
| Diabetes history, n (%) | 860 (8.2) | 1,178 (4.9) | 668 (2.7) |
| High blood pressure history, n (%) | 3,928 (37.4) | 6,046 (25.2) | 4,392 (18.0) |
| Systolic blood pressure (mmHg), mean (SD) | 147.9 (22.9) | 141.2 (18.4) | 135.1 (17.6) |
| Diastolic blood pressure (mmHg), mean (SD) | 86.8 (11.9) | 83.1 (9.9) | 79.3 (9.7) |
| Medication for cholesterol, blood pressure, n (%) |  |  |  |
| None of the above | 8,820 (83.7) | 21,341 (88.7) | 22,500 (92.0) |
| Cholesterol lowering medication | 854 (8.1) | 1,451 (6.0) | 1,043 (4.3) |
| Blood pressure medication | 869 (8.2) | 1,264 (5.3) | 926 (3.8) |

BMI body mass index; PA physical activity; MET basal metabolic-equivalent; SD standard deviation; n numbers

# **Table S9.** Cohort characteristics by age and sex-specific tertile of handgrip strength

|  | **Handgrip strength tertiles** | | |
| --- | --- | --- | --- |
|  | **Lower** | **Middle** | **Higher** |
| **Socio-demographics** |  |  |  |
| Total n | 128,076 | 131,252 | 130,251 |
| Women, n (%) | 71,070 (55.5) | 69,681 (53.1) | 71,804 (55.1) |
| Age (years), mean (SD) | 56.3 (8.0) | 55.9 (8.1) | 55.5 (8.2) |
| Deprivation index quintiles, n (%) |  |  |  |
| Lower | 39,347 (30.7) | 46,246 (35.2) | 48,783 (37.5) |
| Middle | 42,022 (32.8) | 44,802 (34.1) | 44,768 (34.4) |
| Higher | 46,707 (36.5) | 40,204 (30.6) | 36,700 (28.2) |
| Professional qualifications, n (%) |  |  |  |
| College or University degree | 39,255 (38.3) | 44,725 (40.4) | 47,378 (42.0) |
| A levels/AS levels or equivalent | 13,826 (13.5) | 14,982 (13.5) | 15,422 (13.7) |
| O levels/GCSEs or equivalent | 27,386 (26.7) | 28,534 (25.8) | 27,577 (24.5) |
| CSEs or equivalent | 7,783 (7.6) | 7,311 (6.6) | 6,881 (6.1) |
| NVQ or HND or HNC or equivalent | 7,934 (7.7) | 8,590 (7.8) | 8,666 (7.7) |
| Other professional qualifications | 6,321 (6.2) | 6,607 (6.0) | 6,809 (6.0) |
| Income categories, n (%) |  |  |  |
| Less than £18,000 | 26,666 (24.7) | 21,313 (18.7) | 18,430 (16.1) |
| £18,000 to £29,999 | 28,264 (26.2) | 28,401 (24.9) | 27,298 (23.9) |
| £30,000 to £51,999 | 27,382 (25.4) | 31,425 (27.6) | 32,655 (28.6) |
| £52,000 to £100,000 | 20,485 (19.0) | 25,820 (22.7) | 28,002 (24.5) |
| Greater than £100,000 | 5,212 (4.8) | 6,921 (6.1) | 7,812 (6.8) |
| Employment status, n (%) |  |  |  |
| In paid employment or self-employed | 74,505 (58.9) | 82,442 (63.3) | 83,906 (64.9) |
| Retired | 40,789 (32.2) | 39,702 (30.5) | 37,849 (29.3) |
| Looking after home and/or family | 3,639 (2.9) | 3,616 (2.8) | 3,839 (3.0) |
| Unable to work because of sickness or disability | 4,054 (3.2) | 1,504 (1.2) | 1,165 (0.9) |
| Unemployed | 2,648 (2.1) | 2,036 (1.6) | 1,573 (1.2) |
| Doing unpaid or voluntary work | 591 (0.5) | 538 (0.4) | 598 (0.5) |
| Full or part-time student | 361 (0.3) | 352 (0.3) | 373 (0.3) |
| Ethnicity, n (%) |  |  |  |
| White | 117,243 (91.5) | 125,082 (95.3) | 125,085 (96.0) |
| South Asian | 5,210 (4.1) | 1,952 (1.5) | 832 (0.6) |
| Black | 2,321 (1.8) | 1,984 (1.5) | 2,444 (1.9) |
| Chinese | 684 (0.5) | 455 (0.4) | 252 (0.2) |
| Mixed background / others | 2,618 (2.0) | 1,779 (1.4) | 1,638 (1.3) |
| Smoking status, n (%) |  |  |  |
| Never | 74,721 (58.6) | 74,525 (57.0) | 72,425 (55.8) |
| Previous | 40,173 (31.5) | 43,633 (33.4) | 44,981 (34.6) |
| Current | 12,642 (9.9) | 12,694 (9.7) | 12,477 (9.6) |
| **Obesity-related markers** |  |  |  |
| BMI, mean (SD) | 27.3 (4.9) | 27.0 (4.5) | 27.3 (4.5) |
| BMI Categories, n (%) |  |  |  |
| Underweight (<18.5) | 869 (0.7) | 608 (0.5) | 416 (0.3) |
| Normal weight (18.5-24.9) | 43,491 (34.1) | 46,132 (35.2) | 42,138 (32.4) |
| Overweight (25.0 to 29.9) | 52,904 (41.4) | 56,168 (42.9) | 57,382 (44.1) |
| Obese (≥30.0) | 30,402 (23.8) | 28,182 (21.5) | 30,195 (23.2) |
| Waist Circumference (cm), mean (SD) | 89.9 (13.5) | 89.3 (13.1) | 89.7 (13.0) |
| Central Obesity, n (%) | 42,504 (33.2) | 39,062 (29.8) | 41,230 (31.7) |
| % Body fat, mean (SD) | 31.8 (8.6) | 30.8 (8.4) | 30.8 (8.4) |
| **Fitness and Physical activity**, mean (SD) |  |  |  |
| Fitness (METs) | 8.6 (3.5) | 9.2 (3.4) | 9.3 (3.4) |
| Grip strength (kg) | 23.2 (8.1) | 31.4 (8.5) | 38.5 (10.5) |
| Total physical activity (MET.h.week^-1^) | 6.0 (8.8) | 6.5 (9.0) | 7.0 (9.3) |
| TV-viewing (h.day^-1^) | 2.8 (1.7) | 2.7 (1.5) | 2.6 (1.5) |
| PC-screen time (h.day^-1^) | 1.2 (1.4) | 1.2 (1.3) | 1.2 (1.3) |
| Screen-time (h.day^-1^) | 4.0 (2.0) | 3.9 (1.9) | 3.8 (1.9) |
| Sleep duration (h.day-1) | 7.1 (1.0) | 7.1 (1.0) | 7.2 (0.9) |
| **Dietary intakes**, mean (SD) |  |  |  |
| Total energy (Kcal.day^-1^) | 2090.2 (655.7) | 2118.0 (637.1) | 2140.1 (646.7) |
| Protein intake (% of TE) | 15.5 (3.7) | 15.5 (3.6) | 15.6 (3.5) |
| Carbohydrates intake (% of TE) | 47.6 (8.3) | 47.1 (8.1) | 46.9 (8.0) |
| Total Fat intake (% of TE) | 32.0 (6.8) | 32.0 (6.7) | 32.2 (6.7) |
| Saturated intake (% of TE) | 12.3 (3.4) | 12.3 (3.3) | 12.4 (3.3) |
| Sugar intake (% of TE) | 22.6 (7.2) | 22.4 (6.9) | 22.4 (6.8) |
| Alcohol intake (% of TE) | 4.9 (6.5) | 5.4 (6.6) | 5.3 (6.4) |
| Red meat intake (portion.week^-1^) | 1.9 (1.5) | 1.9 (1.4) | 1.9 (1.4) |
| Processed meat intake (portion.week^-1^) | 1.9 (1.1) | 1.9 (1.1) | 2.0 (1.1) |
| Vegetable and Fruit intake (grams.day^-1^) | 324.9 (199.2) | 328.1 (190.5) | 335.2 (189.1) |
| Oily fish (portion.week^-1^) | 1.1 (1.0) | 1.1 (1.0) | 1.1 (1.0) |
| **Health status** |  |  |  |
| Diabetes history, n (%) | 7,456 (5.8) | 5,070 (3.9) | 4,148 (3.2) |
| High blood pressure history, n (%) | 33,737 (26.4) | 31,353 (23.9) | 30,628 (23.6) |
| Systolic blood pressure (mmHg), mean (SD) | 138.8 (19.7) | 139.7 (19.6) | 140.9 (19.5) |
| Diastolic blood pressure (mmHg), mean (SD) | 82.0 (10.7) | 82.4 (10.6) | 83.1 (10.6) |
| Medication for cholesterol, blood pressure, n (%) |  |  |  |
| None of the above | 111,833 (87.3) | 118,102 (90.0) | 117,276 (90.0) |
| Cholesterol lowering medication | 8,395 (6.6) | 6,493 (5.0) | 6,224 (4.8) |
| Blood pressure medication | 7,848 (6.1) | 6,657 (5.1) | 6,751 (5.2) |

BMI body mass index; PA physical activity; MET basal metabolic-equivalent; SD standard deviation; n numbers

# **Table S10.** Correlation between TV viewing, total physical activity and grip strength.

|  | Screen-time  (h.day^-1^) | TV-viewing  (h.day^-1^) | PC-screen  (h.day^-1^) | Total PA  (MET.h.week^-1^) | Grip strength  (Kg) |
| --- | --- | --- | --- | --- | --- |
| Screen-time (h.day^-1^) | -- |  |  |  |  |
| TV-viewing (h.day^-1^) | 0.734* | -- |  |  |  |
| PC-screen (h.day^-1^) | 0.622* | -0.072* | -- |  |  |
| Total PA (MET.h.week^-1^) | -0.059* | -0.021* | -0.068* | -- |  |
| Grip strength (kg) | 0.024* | -0.073* | 0.115* | 0.094* | -- |
| Fitness (METs) | -0.096* | -0.199* | 0.067* | 0.102* | 0.361* |

Data presented as person correlation coefficient. Significant coefficients (p<0.0001) were denoted as *


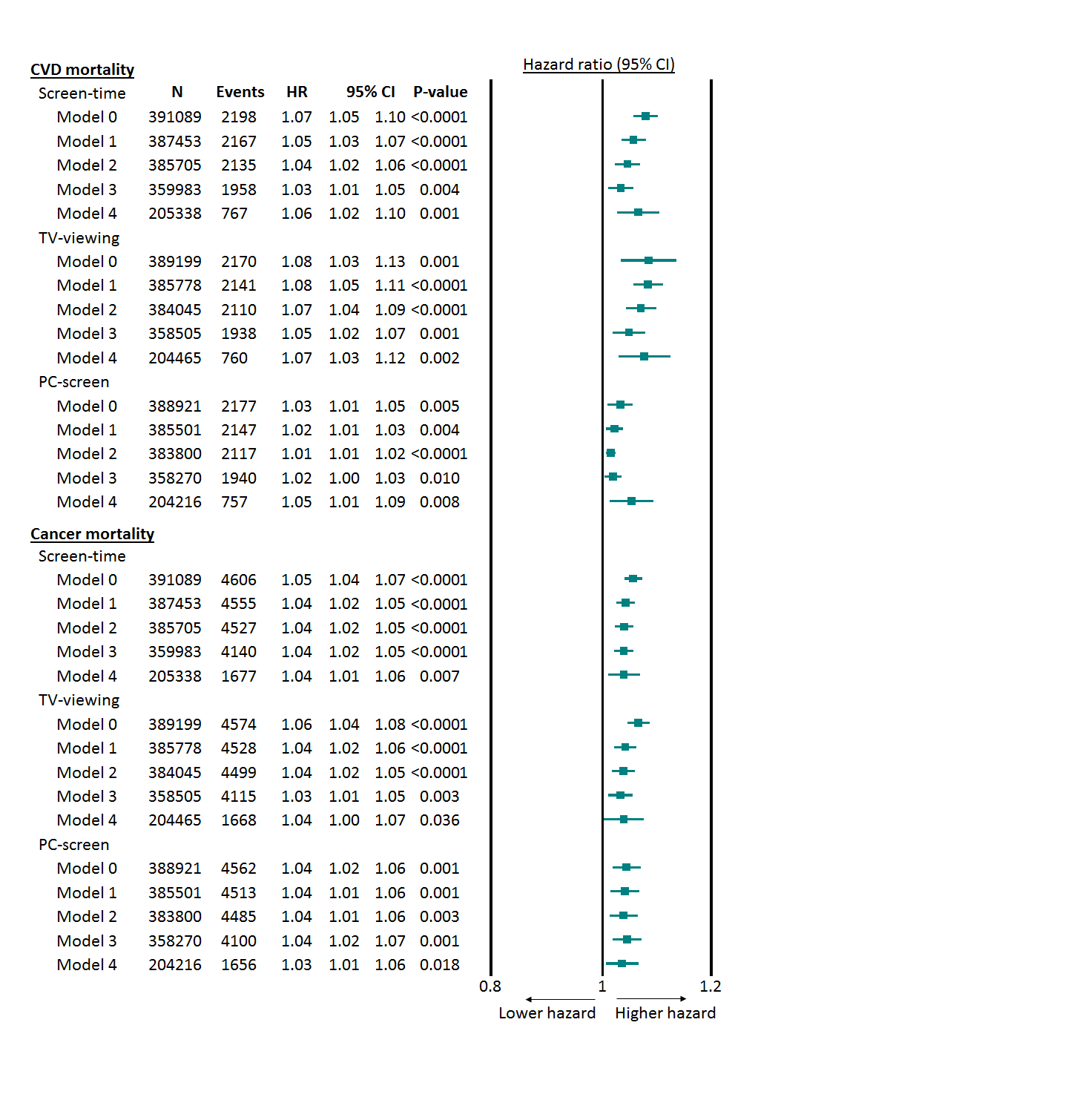


**Figure S1.** Cox proportional hazard model of the association of 1-hour increment in screen-time, TV-viewing and PC-screen with CVD and cancer mortality

Data presented as adjusted hazard ratio (HR) (95%CI) per 1-hour increment in screen-time, TV-viewing and PC-screen time per day. Main outcomes were defined as CVD and cancer mortality. All-analysis were performed as a landmark analysis with follow-up commenced two years after recruitment and including participants who were event-free at this time. Participants with comorbidities at baseline were excluded from all-analysis (depression, COPD, chronic asthma, chronic liver diseases, alcohol problems, substance abuse, eating disorders, schizophrenia, cognitive impartment, Parkinson, dementia, chronic pain syndrome, heart diseases, and cancer (n= 103,755). For these analyses, we ran five incremental models that included an increasing number of covariates: “model 0” included age, sex, ethnicity, deprivation index, professional qualifications, income and employment as covariates; “model 1”, was also adjusted for lifestyle factors including smoking, physical activity, grip strength, sleep duration categories, dietary intake (alcohol, fruit and vegetable, red meat, processed meat and oily fish intake); “model 2” was adjusted for model 1 plus BMI categories; “model 3” was adjusted for model 2 plus systolic blood pressure, prevalent diabetes, hypertension and medication for diabetes, hypertension, and cholesterol. Finally, “model 4” was equivalent to “model 3” but participants who reported been ex-smokers (n=173,104) or current smokers (n=52,990) were excluded from the analysis.


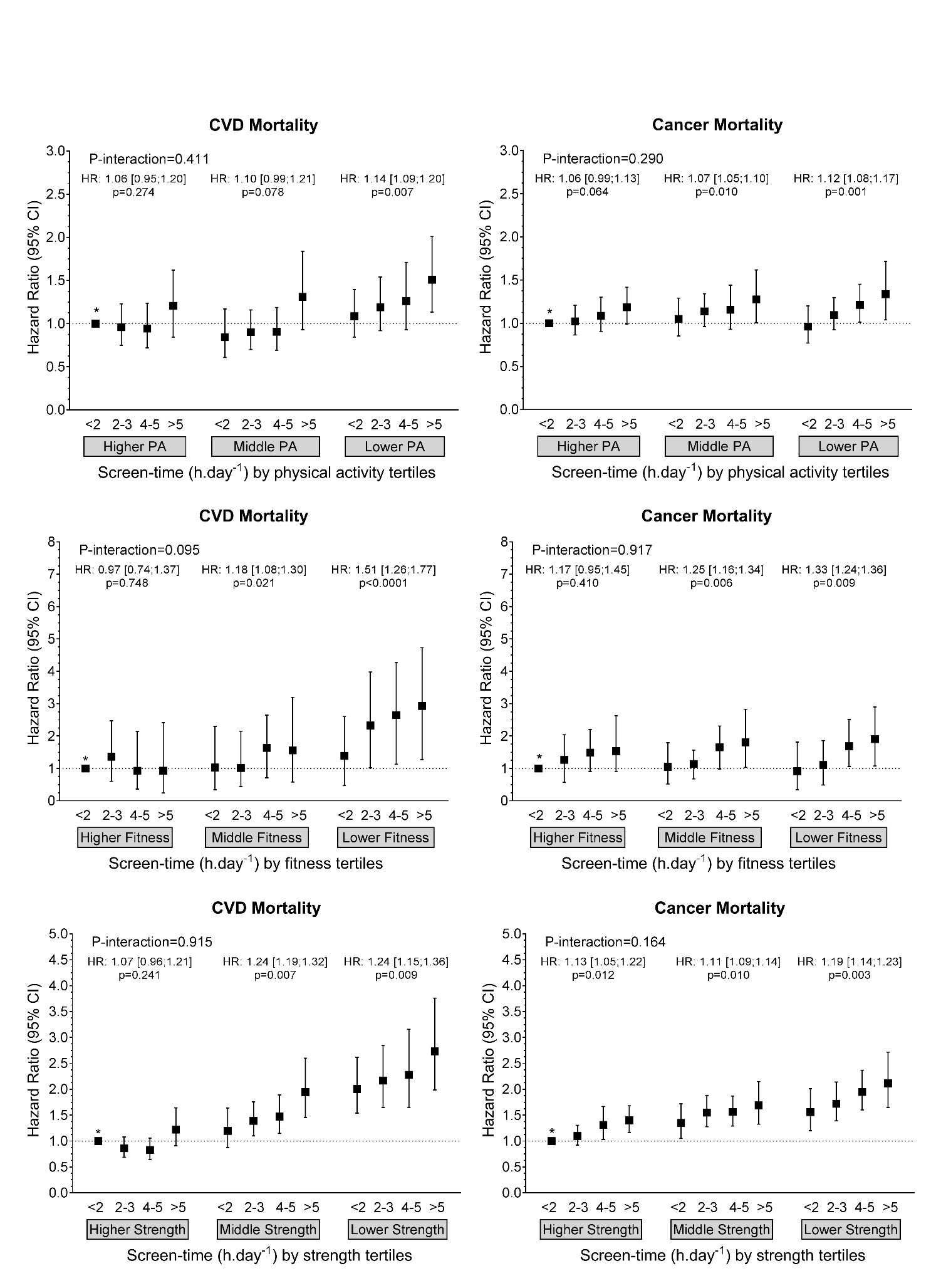


**Figure S2.** Cox proportional hazard models of the association of overall discretionary screen-time with CVD and cancer mortality by physical activity, fitness and handgrip strength strata.

Data presented as adjusted hazard ratio (HR) (95%CI). Reference category was defined as those participants with <2 h.day-1 of screen-time and who were in the highest tertile for physical activity, fitness or grip strength. Analyses were adjusted for age, sex, ethnicity, deprivation index, professional qualifications, income, employment, smoking status, sleep duration categories, dietary intake (alcohol, red meat, processed meat, fruit and vegetable and oily fish intake), systolic blood pressure, prevalent diabetes, hypertension and medication for diabetes, hypertension, and cholesterol. The analysis was performed as a 2-years landmark. Participants with comorbidities at baseline were excluded from all-analysis (n= 103,755). P-interaction indicates the p-value for the interaction between screen-time and tertile of physical activity, fitness or strength.


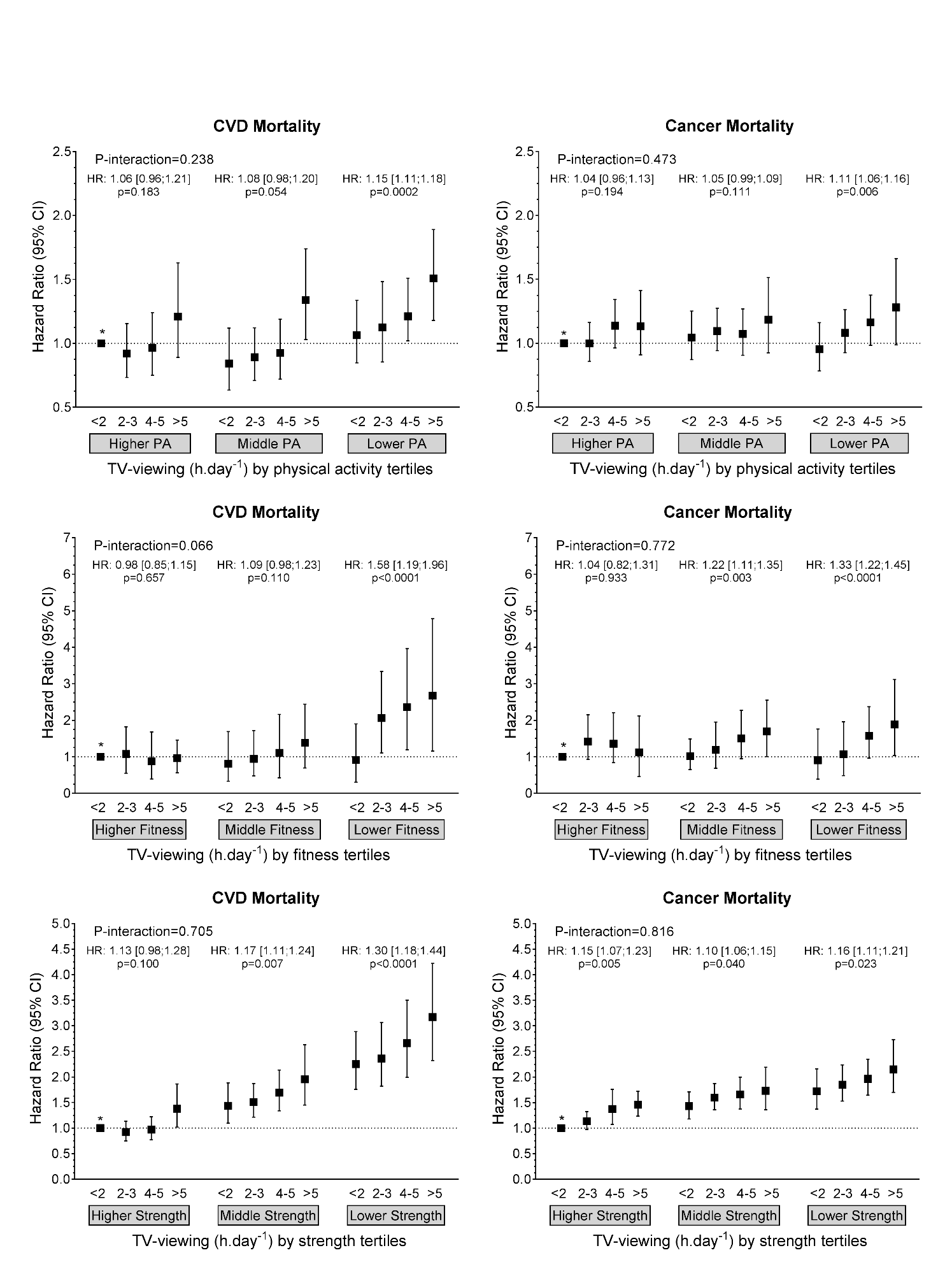


**Figure S3.** Cox proportional hazard models of the association of overall discretionary TV-viewing with CVD and cancer mortality by physical activity, fitness and handgrip strength strata.

Data presented as adjusted hazard ratio (HR) (95%CI). Reference category was defined as those participants with <2 h.day-1 of TV-viewing and who were in the highest tertile for physical activity, fitness or grip strength. Analyses were adjusted for age, sex, ethnicity, deprivation index, professional qualifications, income, employment, smoking status, sleep duration categories, dietary intake (alcohol, red meat, processed meat, fruit and vegetable and oily fish intake), systolic blood pressure, prevalent diabetes, hypertension and medication for diabetes, hypertension, and cholesterol. The analysis was performed as a 2-years landmark. Participants with comorbidities at baseline were excluded from all-analysis (n= 103,755). P-interaction indicates the p-value for the interaction between TV-viewing and tertile of physical activity, fitness or strength.


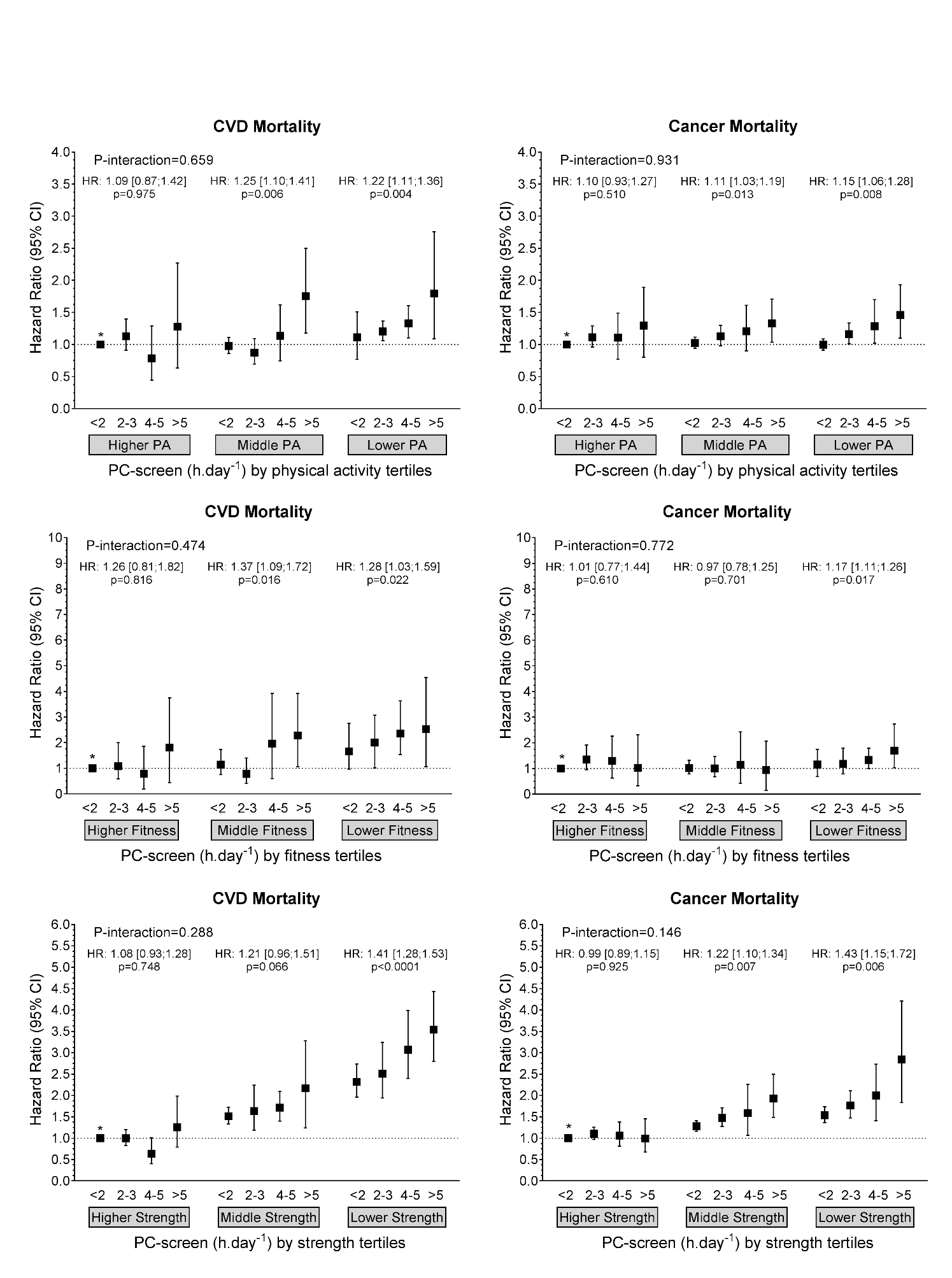


**Figure S4.** Cox proportional hazard models of the association of overall discretionary PC-screen time with CVD and cancer mortality by physical activity, fitness and handgrip strength strata.

Data presented as adjusted hazard ratio (HR) (95%CI). Reference category was defined as those participants with <2 h.day-1 of PC-screen time and who were in the highest tertile for physical activity, fitness or grip strength. Analyses were adjusted for age, sex, ethnicity, deprivation index, professional qualifications, income, employment, smoking status, sleep duration categories, dietary intake (alcohol, red meat, processed meat, fruit and vegetable and oily fish intake), systolic blood pressure, prevalent diabetes, hypertension and medication for diabetes, hypertension, and cholesterol. The analysis was performed as a 2-years landmark. Participants with comorbidities at baseline were excluded from all-analysis (n= 103,755). P-interaction indicates the p-value for the interaction between PC-screen time and tertile of physical activity, fitness or strength.

**Table S11.** Cox proportional hazard estimates of the association of overall discretionary screen-time with all-cause mortality, CVD and cancer incidence and mortality by physical activity, fitness and handgrip strength strata.

|  | **Overall discretionary screen-time categories** | | | | | | | | | | | |
| --- | --- | --- | --- | --- | --- | --- | --- | --- | --- | --- | --- | --- |
|  | <2 h.day^-1^) | | | 2-3 h.day^-1^) | | | 4-5 h.day^-1^) | | | >5 h.day^-1^) | | |
| **Tertiles of PA** | HR | 95% CI | | HR | 95% CI | | HR | 95% CI | | HR | 95% CI | |
| **All-cause mortality** |  | Lower | Upper |  | Lower | Upper |  | Lower | Upper |  | Lower | Upper |
| Higher PA | 1 (Ref.) | | | 0.99 | 0.87 | 1.13 | 1.06 | 0.92 | 1.23 | 1.20 | 0.97 | 1.49 |
| Middle PA | 1.04 | 0.94 | 1.18 | 1.05 | 0.92 | 1.20 | 1.10 | 0.96 | 1.27 | 1.27 | 1.06 | 1.51 |
| Lower PA | 1.04 | 0.88 | 1.24 | 1.10 | 0.97 | 1.26 | 1.26 | 1.09 | 1.44 | 1.41 | 1.18 | 1.69 |
| **CVD mortality** | | | | | | | | | | | | |
| Higher PA | 1 (Ref.) | | | 0.96 | 0.75 | 1.23 | 0.94 | 0.72 | 1.24 | 1.21 | 0.84 | 1.62 |
| Middle PA | 0.84 | 0.61 | 1.17 | 0.90 | 0.70 | 1.16 | 0.91 | 0.69 | 1.19 | 1.31 | 0.93 | 1.84 |
| Lower PA | 1.09 | 0.84 | 1.39 | 1.19 | 0.92 | 1.54 | 1.26 | 0.93 | 1.71 | 1.51 | 1.13 | 2.01 |
| **CVD incidence** | | | | | | | | | | | | |
| Higher PA | 1 (Ref.) | | | 0.97 | 0.88 | 1.07 | 1.02 | 0.94 | 1.09 | 1.03 | 0.95 | 1.11 |
| Middle PA | 0.97 | 0.90 | 1.07 | 0.98 | 0.91 | 1.06 | 1.02 | 0.94 | 1.11 | 1.10 | 1.01 | 1.20 |
| Lower PA | 1.06 | 0.99 | 1.14 | 1.09 | 1.01 | 1.18 | 1.11 | 1.05 | 1.17 | 1.18 | 1.07 | 1.30 |
| **Cancer mortality** | | | | | | | | | | | | |
| Higher PA | 1 (Ref.) | | | 1.02 | 0.86 | 1.21 | 1.09 | 0.91 | 1.30 | 1.19 | 0.99 | 1.42 |
| Middle PA | 1.05 | 0.85 | 1.29 | 1.14 | 0.96 | 1.34 | 1.16 | 0.93 | 1.44 | 1.28 | 1.01 | 1.62 |
| Lower PA | 0.96 | 0.77 | 1.20 | 1.09 | 0.92 | 1.30 | 1.21 | 1.01 | 1.45 | 1.34 | 1.04 | 1.72 |
| **Cancer incidence** | | | | | | | | | | | | |
| Higher PA | 1 (Ref.) | | | 1.05 | 0.98 | 1.13 | 1.08 | 1.00 | 1.16 | 1.12 | 1.01 | 1.23 |
| Middle PA | 0.97 | 0.89 | 1.05 | 1.07 | 1.00 | 1.15 | 1.12 | 1.04 | 1.21 | 1.17 | 1.05 | 1.30 |
| Lower PA | 0.96 | 0.87 | 1.05 | 1.06 | 0.99 | 1.14 | 1.16 | 1.08 | 1.25 | 1.23 | 1.09 | 1.38 |
| **Tertiles of fitness** | | | | | | | | | | | | |
| **All-cause mortality** | | | | | | | | | | | | |
| Higher fitness | 1 (Ref.) | | | 1.21 | 0.83 | 1.76 | 1.19 | 0.79 | 1.81 | 1.20 | 0.66 | 1.88 |
| Middle fitness | 0.93 | 0.55 | 1.44 | 0.98 | 0.67 | 1.41 | 1.31 | 0.88 | 1.90 | 1.42 | 0.90 | 1.98 |
| Lower fitness | 1.26 | 0.72 | 2.00 | 1.60 | 1.02 | 2.40 | 1.71 | 1.13 | 2.58 | 1.98 | 1.30 | 2.92 |
| **CVD mortality** | | | | | | | | | | | | |
| Higher fitness | 1 (Ref.) | | | 1.36 | 0.60 | 2.47 | 0.93 | 0.37 | 2.14 | 0.93 | 0.24 | 2.42 |
| Middle fitness | 1.03 | 0.34 | 2.30 | 1.02 | 0.44 | 2.15 | 1.63 | 0.71 | 2.65 | 1.56 | 0.58 | 3.20 |
| Lower fitness | 1.39 | 0.47 | 2.60 | 2.33 | 1.02 | 3.98 | 2.65 | 1.13 | 4.28 | 2.93 | 1.27 | 4.73 |
| **CVD incidence** | | | | | | | | | | | | |
| Higher fitness | 1 (Ref.) | | | 1.06 | 0.87 | 1.28 | 0.95 | 0.76 | 1.18 | 0.85 | 0.60 | 1.19 |
| Middle fitness | 0.91 | 0.70 | 1.19 | 0.85 | 0.69 | 1.05 | 1.00 | 0.83 | 1.22 | 1.02 | 0.78 | 1.34 |
| Lower fitness | 1.30 | 0.98 | 1.72 | 1.51 | 1.21 | 1.89 | 1.71 | 1.39 | 2.10 | 1.79 | 1.36 | 2.37 |
| **Cancer mortality** | | | | | | | | | | | | |
| Higher fitness | 1 (Ref.) | | | 1.26 | 0.57 | 2.04 | 1.49 | 0.91 | 2.20 | 1.54 | 0.90 | 2.63 |
| Middle fitness | 1.05 | 0.52 | 1.80 | 1.14 | 0.68 | 1.57 | 1.66 | 0.98 | 2.31 | 1.81 | 1.03 | 2.83 |
| Lower fitness | 0.92 | 0.34 | 1.81 | 1.11 | 0.49 | 1.86 | 1.69 | 1.06 | 2.51 | 1.91 | 1.07 | 2.90 |
| **Cancer incidence** | | | | | | | | | | | | |
| Higher fitness | 1 (Ref.) | | | 0.98 | 0.83 | 1.16 | 0.99 | 0.81 | 1.19 | 0.99 | 0.74 | 1.33 |
| Middle fitness | 1.03 | 0.82 | 1.30 | 0.93 | 0.78 | 1.10 | 0.98 | 0.81 | 1.18 | 0.95 | 0.74 | 1.23 |
| Lower fitness | 0.86 | 0.70 | 1.04 | 1.01 | 0.83 | 1.24 | 1.11 | 0.91 | 1.37 | 1.23 | 1.02 | 1.57 |
| **Tertiles of handgrip strength** | | | | | | | | | | | | |
| **All-cause mortality** | | | | | | | | | | | | |
| Higher strength | 1 (Ref.) | | | 1.00 | 0.87 | 1.14 | 1.17 | 1.02 | 1.35 | 1.30 | 1.09 | 1.56 |
| Middle strength | 1.58 | 1.33 | 1.86 | 1.55 | 1.36 | 1.78 | 1.62 | 1.40 | 1.88 | 1.92 | 1.61 | 2.30 |
| Lower strength | 2.06 | 1.71 | 2.47 | 2.26 | 1.94 | 2.62 | 2.31 | 1.97 | 2.70 | 2.84 | 2.36 | 3.42 |
| **CVD mortality** | | | | | | | | | | | | |
| Higher strength | 1 (Ref.) | | | 0.86 | 0.69 | 1.08 | 0.83 | 0.65 | 1.06 | 1.22 | 0.91 | 1.64 |
| Middle strength | 1.20 | 0.87 | 1.64 | 1.39 | 1.10 | 1.76 | 1.47 | 1.15 | 1.89 | 1.94 | 1.45 | 2.60 |
| Lower strength | 2.01 | 1.54 | 2.62 | 2.17 | 1.65 | 2.85 | 2.28 | 1.64 | 3.16 | 2.73 | 1.98 | 3.76 |
| **CVD incidence** | | | | | | | | | | | | |
| Higher strength | 1 (Ref.) | | | 1.12 | 1.05 | 1.21 | 1.17 | 1.08 | 1.26 | 1.17 | 1.06 | 1.30 |
| Middle strength | 1.38 | 1.25 | 1.51 | 1.40 | 1.30 | 1.51 | 1.47 | 1.36 | 1.60 | 1.68 | 1.52 | 1.87 |
| Lower strength | 1.58 | 1.43 | 1.74 | 1.76 | 1.59 | 1.96 | 1.79 | 1.65 | 1.95 | 2.00 | 1.79 | 2.24 |
| **Cancer mortality** | | | | | | | | | | | | |
| Higher strength | 1 (Ref.) | | | 1.10 | 0.92 | 1.30 | 1.31 | 1.03 | 1.67 | 1.40 | 1.17 | 1.68 |
| Middle strength | 1.35 | 1.05 | 1.72 | 1.55 | 1.28 | 1.88 | 1.56 | 1.29 | 1.87 | 1.69 | 1.33 | 2.15 |
| Lower strength | 1.56 | 1.20 | 2.01 | 1.72 | 1.39 | 2.14 | 1.95 | 1.60 | 2.37 | 2.12 | 1.65 | 2.72 |
| **Cancer incidence** | | | | | | | | | | | | |
| Higher strength | 1 (Ref.) | | | 1.15 | 1.07 | 1.24 | 1.26 | 1.12 | 1.40 | 1.29 | 1.19 | 1.39 |
| Middle strength | 1.28 | 1.17 | 1.41 | 1.39 | 1.25 | 1.50 | 1.45 | 1.34 | 1.58 | 1.48 | 1.33 | 1.66 |
| Lower strength | 1.40 | 1.26 | 1.55 | 1.45 | 1.33 | 1.60 | 1.53 | 1.41 | 1.66 | 1.67 | 1.49 | 1.87 |

Data presented as adjusted hazard ratio (HR) (95%CI). Reference category was defined as those participants with <2 h.day-1 of screen-time and who were in the highest tertile for physical activity, fitness or grip strength. Analyses were adjusted for age, sex, ethnicity, deprivation index, professional qualifications, income, employment, smoking status, sleep duration categories, dietary intake (alcohol, red meat, processed meat, fruit and vegetable and oily fish intake), systolic blood pressure, prevalent diabetes, hypertension and medication for diabetes, hypertension, and cholesterol. The analysis was performed as a 2-years landmark. Participants with comorbidities at baseline were excluded from all-analysis (n= 103,755). P-interaction indicates the p-value for the interaction between screen-time and tertile of physical activity, fitness or strength.

**Table S12.** Cox proportional hazard estimates of the association of discretionary TV-viewing with all-cause mortality, CVD and cancer incidence and mortality by physical activity, fitness and handgrip strength strata.

|  | **TV-viewing categories** | | | | | | | | | | | |
| --- | --- | --- | --- | --- | --- | --- | --- | --- | --- | --- | --- | --- |
|  | <2 h.day^-1^) | | | 2-3 h.day^-1^) | | | 4-5 h.day^-1^) | | | >5 h.day^-1^) | | |
| **Tertiles of PA** | HR | 95% CI | | HR | 95% CI | | HR | 95% CI | | HR | 95% CI | |
| **All-cause mortality** |  | Lower | Upper |  | Lower | Upper |  | Lower | Upper |  | Lower | Upper |
| Higher PA | 1 (Ref.) | | | 0.99 | 0.87 | 1.11 | 1.09 | 0.95 | 1.24 | 1.17 | 0.96 | 1.41 |
| Middle PA | 1.02 | 0.88 | 1.18 | 1.05 | 0.93 | 1.18 | 1.10 | 0.96 | 1.26 | 1.28 | 1.14 | 1.44 |
| Lower PA | 1.03 | 0.93 | 1.13 | 1.10 | 1.01 | 1.21 | 1.25 | 1.10 | 1.43 | 1.40 | 1.19 | 1.65 |
| **CVD mortality** | | | | | | | | | | | | |
| Higher PA | 1 (Ref.) | | | 0.92 | 0.73 | 1.15 | 0.96 | 0.75 | 1.24 | 1.21 | 0.89 | 1.63 |
| Middle PA | 0.84 | 0.63 | 1.12 | 0.89 | 0.71 | 1.12 | 0.93 | 0.72 | 1.19 | 1.34 | 1.03 | 1.74 |
| Lower PA | 1.06 | 0.85 | 1.34 | 1.13 | 0.85 | 1.48 | 1.21 | 1.02 | 1.51 | 1.51 | 1.18 | 1.89 |
| **CVD incidence** | | | | | | | | | | | | |
| Higher PA | 1 (Ref.) | | | 0.98 | 0.90 | 1.07 | 1.02 | 0.95 | 1.09 | 1.02 | 0.95 | 1.10 |
| Middle PA | 0.98 | 0.90 | 1.05 | 0.98 | 0.92 | 1.05 | 1.02 | 0.94 | 1.10 | 1.10 | 1.01 | 1.22 |
| Lower PA | 1.05 | 0.98 | 1.13 | 1.08 | 1.00 | 1.17 | 1.09 | 1.01 | 1.18 | 1.17 | 1.06 | 1.29 |
| **Cancer mortality** | | | | | | | | | | | | |
| Higher PA | 1 (Ref.) | | | 1.00 | 0.86 | 1.16 | 1.14 | 0.96 | 1.34 | 1.13 | 0.91 | 1.41 |
| Middle PA | 1.04 | 0.87 | 1.25 | 1.10 | 0.94 | 1.27 | 1.07 | 0.91 | 1.27 | 1.18 | 0.93 | 1.51 |
| Lower PA | 0.95 | 0.78 | 1.16 | 1.08 | 0.93 | 1.26 | 1.16 | 0.98 | 1.38 | 1.28 | 0.99 | 1.66 |
| **Cancer incidence** | | | | | | | | | | | | |
| Higher PA | 1 (Ref.) | | | 1.03 | 0.97 | 1.10 | 1.06 | 0.99 | 1.14 | 1.10 | 0.99 | 1.22 |
| Middle PA | 0.97 | 0.90 | 1.04 | 1.05 | 0.99 | 1.12 | 1.11 | 1.03 | 1.19 | 1.15 | 1.02 | 1.29 |
| Lower PA | 0.95 | 0.88 | 1.03 | 1.05 | 0.98 | 1.11 | 1.13 | 1.05 | 1.22 | 1.24 | 1.10 | 1.40 |
| **Tertiles of fitness** | | | | | | | | | | | | |
| **All-cause mortality** | | | | | | | | | | | | |
| Higher fitness | 1 (Ref.) | | | 1.15 | 0.83 | 1.59 | 1.05 | 0.72 | 1.54 | 1.11 | 0.57 | 1.78 |
| Middle fitness | 0.91 | 0.59 | 1.41 | 0.92 | 0.66 | 1.29 | 1.23 | 0.87 | 1.75 | 1.34 | 0.83 | 1.96 |
| Lower fitness | 1.02 | 0.57 | 1.62 | 1.54 | 1.16 | 2.02 | 1.78 | 1.31 | 2.41 | 1.91 | 1.32 | 2.72 |
| **CVD mortality** | | | | | | | | | | | | |
| Higher fitness | 1 (Ref.) | | | 1.08 | 0.55 | 1.82 | 0.88 | 0.39 | 1.68 | 0.96 | 0.56 | 1.46 |
| Middle fitness | 0.81 | 0.33 | 1.69 | 0.94 | 0.48 | 1.72 | 1.11 | 0.42 | 2.16 | 1.38 | 0.69 | 2.44 |
| Lower fitness | 0.91 | 0.31 | 1.90 | 2.06 | 1.10 | 3.34 | 2.36 | 1.19 | 3.96 | 2.68 | 1.16 | 4.78 |
| **CVD incidence** | | | | | | | | | | | | |
| Higher fitness | 1 (Ref.) | | | 1.11 | 0.93 | 1.31 | 0.99 | 0.81 | 1.22 | 0.86 | 0.57 | 1.19 |
| Middle fitness | 0.96 | 0.77 | 1.16 | 1.06 | 0.89 | 1.26 | 0.87 | 0.71 | 1.06 | 1.05 | 0.80 | 1.36 |
| Lower fitness | 1.24 | 0.98 | 1.56 | 1.54 | 1.25 | 1.89 | 1.78 | 1.48 | 2.14 | 1.87 | 1.56 | 2.28 |
| **Cancer mortality** | | | | | | | | | | | | |
| Higher fitness | 1 (Ref.) | | | 1.42 | 0.93 | 2.15 | 1.36 | 0.84 | 2.21 | 1.12 | 0.46 | 2.12 |
| Middle fitness | 1.02 | 0.65 | 1.49 | 1.19 | 0.69 | 1.95 | 1.50 | 0.95 | 2.27 | 1.69 | 1.01 | 2.55 |
| Lower fitness | 0.91 | 0.39 | 1.76 | 1.07 | 0.49 | 1.96 | 1.57 | 0.96 | 2.37 | 1.88 | 1.04 | 3.12 |
| **Cancer incidence** | | | | | | | | | | | | |
| Higher fitness | 1 (Ref.) | | | 1.01 | 0.87 | 1.18 | 0.99 | 0.82 | 1.19 | 1.05 | 0.82 | 1.36 |
| Middle fitness | 1.03 | 0.85 | 1.26 | 0.94 | 0.80 | 1.10 | 1.00 | 0.84 | 1.19 | 1.05 | 0.81 | 1.37 |
| Lower fitness | 0.96 | 0.72 | 1.31 | 1.04 | 0.78 | 1.38 | 1.15 | 0.96 | 1.36 | 1.26 | 1.05 | 1.51 |
| **Tertiles of handgrip strength** | | | | | | | | | | | | |
| **All-cause mortality** | | | | | | | | | | | | |
| Higher strength | 1 (Ref.) | | | 1.06 | 0.94 | 1.19 | 1.27 | 1.11 | 1.44 | 1.43 | 1.19 | 1.72 |
| Middle strength | 1.63 | 1.44 | 1.85 | 1.70 | 1.47 | 1.97 | 1.71 | 1.50 | 1.96 | 1.94 | 1.62 | 2.32 |
| Lower strength | 2.25 | 1.90 | 2.65 | 2.36 | 2.06 | 2.71 | 2.43 | 2.10 | 2.82 | 3.00 | 2.54 | 3.49 |
| **CVD mortality** | | | | | | | | | | | | |
| Higher strength | 1 (Ref.) | | | 0.92 | 0.75 | 1.13 | 0.97 | 0.77 | 1.22 | 1.38 | 1.02 | 1.86 |
| Middle strength | 1.44 | 1.10 | 1.88 | 1.51 | 1.22 | 1.87 | 1.69 | 1.34 | 2.14 | 1.95 | 1.45 | 2.63 |
| Lower strength | 2.25 | 1.76 | 2.89 | 2.36 | 1.82 | 3.06 | 2.66 | 1.99 | 3.50 | 3.17 | 2.32 | 4.22 |
| **CVD incidence** | | | | | | | | | | | | |
| Higher strength | 1 (Ref.) | | | 1.08 | 1.02 | 1.15 | 1.14 | 1.06 | 1.22 | 1.10 | 0.98 | 1.23 |
| Middle strength | 1.23 | 1.13 | 1.34 | 1.37 | 1.29 | 1.47 | 1.41 | 1.31 | 1.52 | 1.61 | 1.44 | 1.79 |
| Lower strength | 1.46 | 1.32 | 1.61 | 1.63 | 1.47 | 1.80 | 1.75 | 1.62 | 1.89 | 1.94 | 1.74 | 2.17 |
| **Cancer mortality** | | | | | | | | | | | | |
| Higher strength | 1 (Ref.) | | | 1.13 | 0.97 | 1.32 | 1.37 | 1.07 | 1.76 | 1.46 | 1.24 | 1.72 |
| Middle strength | 1.43 | 1.18 | 1.71 | 1.60 | 1.36 | 1.87 | 1.66 | 1.38 | 2.00 | 1.73 | 1.36 | 2.19 |
| Lower strength | 1.72 | 1.37 | 2.16 | 1.85 | 1.53 | 2.24 | 1.96 | 1.64 | 2.35 | 2.15 | 1.70 | 2.73 |
| **Cancer incidence** | | | | | | | | | | | | |
| Higher strength | 1 (Ref.) | | | 1.12 | 1.05 | 1.19 | 1.27 | 1.18 | 1.36 | 1.28 | 1.13 | 1.44 |
| Middle strength | 1.28 | 1.18 | 1.39 | 1.34 | 1.26 | 1.44 | 1.41 | 1.31 | 1.52 | 1.45 | 1.29 | 1.63 |
| Lower strength | 1.33 | 1.21 | 1.47 | 1.47 | 1.36 | 1.60 | 1.49 | 1.38 | 1.61 | 1.68 | 1.51 | 1.87 |

Data presented as adjusted hazard ratio (HR) (95%CI). Reference category was defined as those participants with <2 h.day-1 of TV-viewing and who were in the highest tertile for physical activity, fitness or grip strength. Analyses were adjusted for age, sex, ethnicity, deprivation index, professional qualifications, income, employment, smoking status, sleep duration categories, dietary intake (alcohol, red meat, processed meat, fruit and vegetable and oily fish intake), systolic blood pressure, prevalent diabetes, hypertension and medication for diabetes, hypertension, and cholesterol. The analysis was performed as a 2-years landmark. Participants with comorbidities at baseline were excluded from all-analysis (n= 103,755). P-interaction indicates the p-value for the interaction between TV-viewing and tertile of physical activity, fitness or strength.

**Table S13.** Cox proportional hazard estimates of the association of discretionary PC-screen time with all-cause mortality, CVD and cancer incidence and mortality by physical activity, fitness and handgrip strength strata.

|  | **PC-screen time categories** | | | | | | | | | | | |
| --- | --- | --- | --- | --- | --- | --- | --- | --- | --- | --- | --- | --- |
|  | <2 h.day^-1^) | | | 2-3 h.day^-1^) | | | 4-5 h.day^-1^) | | | >5 h.day^-1^) | | |
| **Tertiles of PA** | HR | 95% CI | | HR | 95% CI | | HR | 95% CI | | HR | 95% CI | |
| **All-cause mortality** |  | Lower | Upper |  | Lower | Upper |  | Lower | Upper |  | Lower | Upper |
| Higher PA | 1 (Ref.) | | | 1.13 | 1.01 | 1.27 | 1.14 | 0.94 | 1.39 | 1.23 | 1.10 | 1.37 |
| Middle PA | 1.05 | 0.98 | 1.13 | 1.06 | 0.94 | 1.19 | 1.14 | 0.90 | 1.45 | 1.37 | 1.13 | 1.66 |
| Lower PA | 1.11 | 1.04 | 1.19 | 1.32 | 1.06 | 1.61 | 1.40 | 1.13 | 1.76 | 1.67 | 1.25 | 2.12 |
| **CVD mortality** | | | | | | | | | | | | |
| Higher PA | 1 (Ref.) | | | 1.13 | 0.91 | 1.40 | 0.78 | 0.44 | 1.29 | 1.28 | 0.63 | 2.27 |
| Middle PA | 0.98 | 0.86 | 1.11 | 0.87 | 0.70 | 1.09 | 1.13 | 0.75 | 1.62 | 1.75 | 1.18 | 2.50 |
| Lower PA | 1.11 | 0.77 | 1.51 | 1.20 | 1.06 | 1.37 | 1.33 | 1.10 | 1.60 | 1.79 | 1.09 | 2.76 |
| **CVD incidence** | | | | | | | | | | | | |
| Higher PA | 1 (Ref.) | | | 0.95 | 0.89 | 1.01 | 1.04 | 0.91 | 1.19 | 1.16 | 0.95 | 1.42 |
| Middle PA | 0.93 | 0.90 | 0.97 | 1.04 | 0.98 | 1.11 | 1.09 | 0.97 | 1.23 | 1.15 | 1.00 | 1.32 |
| Lower PA | 1.09 | 1.03 | 1.16 | 1.11 | 1.05 | 1.19 | 1.17 | 1.01 | 1.36 | 1.24 | 1.05 | 1.46 |
| **Cancer mortality** | | | | | | | | | | | | |
| Higher PA | 1 (Ref.) | | | 1.11 | 0.96 | 1.29 | 1.11 | 0.77 | 1.49 | 1.29 | 0.80 | 1.89 |
| Middle PA | 1.02 | 0.94 | 1.12 | 1.13 | 0.98 | 1.30 | 1.21 | 0.90 | 1.61 | 1.33 | 1.04 | 1.71 |
| Lower PA | 1.00 | 0.91 | 1.09 | 1.16 | 1.01 | 1.33 | 1.29 | 1.02 | 1.70 | 1.46 | 1.10 | 1.93 |
| **Cancer incidence** | | | | | | | | | | | | |
| Higher PA | 1 (Ref.) | | | 1.04 | 0.98 | 1.11 | 1.04 | 0.92 | 1.18 | 1.02 | 0.86 | 1.20 |
| Middle PA | 0.99 | 0.95 | 1.03 | 1.06 | 1.00 | 1.12 | 1.10 | 0.97 | 1.25 | 1.13 | 0.94 | 1.36 |
| Lower PA | 0.97 | 0.94 | 1.01 | 1.04 | 0.98 | 1.11 | 1.13 | 1.06 | 1.21 | 1.20 | 1.04 | 1.38 |
| **Tertiles of fitness** | | | | | | | | | | | | |
| **All-cause mortality** | | | | | | | | | | | | |
| Higher fitness | 1 (Ref.) | | | 1.19 | 0.89 | 1.59 | 1.07 | 0.64 | 1.58 | 1.38 | 0.91 | 1.95 |
| Middle fitness | 1.02 | 0.82 | 1.25 | 0.90 | 0.66 | 1.24 | 0.98 | 0.66 | 1.41 | 1.57 | 1.05 | 2.18 |
| Lower fitness | 1.38 | 0.98 | 1.80 | 1.54 | 1.22 | 1.96 | 1.79 | 1.28 | 2.33 | 2.09 | 1.39 | 2.85 |
| **CVD mortality** | | | | | | | | | | | | |
| Higher fitness | 1 (Ref.) | | | 1.08 | 0.59 | 2.00 | 0.79 | 0.19 | 1.86 | 1.81 | 0.43 | 3.75 |
| Middle fitness | 1.15 | 0.76 | 1.73 | 0.78 | 0.41 | 1.40 | 1.97 | 0.60 | 3.92 | 2.28 | 1.06 | 3.92 |
| Lower fitness | 1.66 | 0.97 | 2.76 | 2.01 | 1.02 | 3.07 | 2.36 | 1.54 | 3.63 | 2.52 | 1.06 | 4.54 |
| **CVD incidence** | | | | | | | | | | | | |
| Higher fitness | 1 (Ref.) | | | 0.84 | 0.51 | 1.29 | 0.86 | 0.60 | 1.22 | 1.04 | 0.88 | 1.22 |
| Middle fitness | 0.95 | 0.85 | 1.06 | 0.92 | 0.78 | 1.08 | 1.00 | 0.75 | 1.35 | 1.04 | 0.69 | 1.51 |
| Lower fitness | 1.45 | 1.26 | 1.64 | 1.60 | 1.37 | 1.84 | 1.71 | 1.43 | 2.04 | 1.76 | 1.34 | 2.30 |
| **Cancer mortality** | | | | | | | | | | | | |
| Higher fitness | 1 (Ref.) | | | 1.35 | 0.95 | 1.91 | 1.30 | 0.63 | 2.26 | 1.02 | 0.32 | 2.32 |
| Middle fitness | 1.02 | 0.78 | 1.32 | 1.00 | 0.68 | 1.48 | 1.14 | 0.42 | 2.43 | 0.94 | 0.14 | 2.07 |
| Lower fitness | 1.16 | 0.69 | 1.75 | 1.18 | 0.79 | 1.80 | 1.33 | 0.99 | 1.79 | 1.70 | 1.03 | 2.73 |
| **Cancer incidence** | | | | | | | | | | | | |
| Higher fitness | 1 (Ref.) | | | 1.02 | 0.88 | 1.19 | 0.98 | 0.71 | 1.27 | 0.89 | 0.57 | 1.31 |
| Middle fitness | 1.00 | 0.90 | 1.10 | 0.95 | 0.82 | 1.11 | 0.96 | 0.71 | 1.23 | 0.94 | 0.60 | 1.30 |
| Lower fitness | 0.89 | 0.68 | 1.16 | 0.91 | 0.73 | 1.15 | 1.06 | 0.80 | 1.36 | 1.16 | 0.99 | 1.38 |
| **Tertiles of handgrip strength** | | | | | | | | | | | | |
| **All-cause mortality** | | | | | | | | | | | | |
| Higher strength | 1 (Ref.) | | | 1.05 | 0.94 | 1.16 | 0.99 | 0.80 | 1.22 | 1.13 | 0.86 | 1.49 |
| Middle strength | 1.45 | 1.34 | 1.56 | 1.62 | 1.45 | 1.82 | 1.91 | 1.55 | 2.36 | 2.34 | 1.95 | 2.85 |
| Lower strength | 2.08 | 1.89 | 2.28 | 2.32 | 2.02 | 2.67 | 2.68 | 2.34 | 3.04 | 3.04 | 2.55 | 3.65 |
| **CVD mortality** | | | | | | | | | | | | |
| Higher strength | 1 (Ref.) | | | 1.00 | 0.83 | 1.20 | 0.64 | 0.40 | 1.01 | 1.25 | 0.79 | 1.99 |
| Middle strength | 1.52 | 1.33 | 1.73 | 1.64 | 1.19 | 2.24 | 1.71 | 1.40 | 2.10 | 2.17 | 1.24 | 3.28 |
| Lower strength | 2.32 | 1.96 | 2.74 | 2.51 | 1.94 | 3.24 | 3.07 | 2.40 | 3.99 | 3.54 | 2.80 | 4.43 |
| **CVD incidence** | | | | | | | | | | | | |
| Higher strength | 1 (Ref.) | | | 1.12 | 1.06 | 1.18 | 1.03 | 0.92 | 1.15 | 1.16 | 1.01 | 1.34 |
| Middle strength | 1.27 | 1.22 | 1.33 | 1.44 | 1.35 | 1.54 | 1.44 | 1.26 | 1.64 | 1.68 | 1.42 | 1.98 |
| Lower strength | 1.57 | 1.49 | 1.66 | 1.79 | 1.64 | 1.94 | 1.94 | 1.64 | 2.25 | 2.21 | 1.89 | 2.58 |
| **Cancer mortality** | | | | | | | | | | | | |
| Higher strength | 1 (Ref.) | | | 1.10 | 0.97 | 1.25 | 1.06 | 0.81 | 1.38 | 0.99 | 0.68 | 1.45 |
| Middle strength | 1.28 | 1.16 | 1.41 | 1.48 | 1.28 | 1.71 | 1.59 | 1.07 | 2.26 | 1.93 | 1.49 | 2.50 |
| Lower strength | 1.54 | 1.36 | 1.74 | 1.76 | 1.48 | 2.11 | 2.00 | 1.41 | 2.73 | 2.84 | 1.83 | 4.21 |
| **Cancer incidence** | | | | | | | | | | | | |
| Higher strength | 1 (Ref.) | | | 1.06 | 1.00 | 1.12 | 1.00 | 0.89 | 1.13 | 0.96 | 0.82 | 1.14 |
| Middle strength | 1.16 | 1.11 | 1.22 | 1.21 | 1.10 | 1.31 | 1.31 | 1.23 | 1.40 | 1.36 | 1.19 | 1.54 |
| Lower strength | 1.27 | 1.20 | 1.34 | 1.42 | 1.31 | 1.54 | 1.59 | 1.45 | 1.75 | 1.70 | 1.51 | 1.92 |

Data presented as adjusted hazard ratio (HR) (95%CI). Reference category was defined as those participants with <2 h.day-1 of PC-screen time and who were in the highest tertile for physical activity, fitness or grip strength. Analyses were adjusted for age, sex, ethnicity, deprivation index, professional qualifications, income, employment, smoking status, sleep duration categories, dietary intake (alcohol, red meat, processed meat, fruit and vegetable and oily fish intake), systolic blood pressure, prevalent diabetes, hypertension and medication for diabetes, hypertension, and cholesterol. The analysis was performed as a 2-years landmark. Participants with comorbidities at baseline were excluded from all-analysis (n= 103,755). P-interaction indicates the p-value for the interaction between PC-screen time and tertile of physical activity, fitness or strength.
